# A Single-Stranded Oligonucleotide Inhibits Toll-Like Receptor 3 Activation and Reduces Influenza A (H1N1) Infection

**DOI:** 10.3389/fimmu.2019.02161

**Published:** 2019-09-12

**Authors:** Candice Poux, Aleksandra Dondalska, Joseph Bergenstråhle, Sandra Pålsson, Vanessa Contreras, Claudia Arasa, Peter Järver, Jan Albert, David C. Busse, Roger LeGrand, Joakim Lundeberg, John S. Tregoning, Anna-Lena Spetz

**Affiliations:** ^1^Department of Molecular Biosciences, The Wenner-Gren Institute, Stockholm University, Stockholm, Sweden; ^2^Science for Life Laboratory, Department of Gene Technology, Royal Institute of Technology, Stockholm, Sweden; ^3^CEA, UMR1184, IDMIT Department, Institut de Biologie François Jacob, DRF, Fontenay-aux-Roses, France; ^4^Department of Microbiology, Tumor and Cell Biology, Karolinska Institutet, Stockholm, Sweden; ^5^Department of Clinical Microbiology, Karolinska University Hospital, Stockholm, Sweden; ^6^Department of Infectious Disease, Imperial College London, London, United Kingdom

**Keywords:** influenza A, TLR3, single-stranded oligonucleotides, human monocyte-derived dendritic cells (MoDC), mice, cytokines, co-stimulatory molecules, clathrin-mediated endocytosis

## Abstract

The initiation of an immune response is dependent on the activation and maturation of dendritic cells after sensing pathogen associated molecular patterns by pattern recognition receptors. However, the response needs to be balanced as excessive pro-inflammatory cytokine production in response to viral or stress-induced pattern recognition receptor signaling has been associated with severe influenza A virus (IAV) infection. Here, we use an inhibitor of Toll-like receptor (TLR)3, a single-stranded oligonucleotide (ssON) with the capacity to inhibit certain endocytic routes, or a TLR3 agonist (synthetic double-stranded RNA PolyI:C), to evaluate modulation of innate responses during H1N1 IAV infection. Since IAV utilizes cellular endocytic machinery for viral entry, we also assessed ssON's capacity to affect IAV infection. We first show that IAV infected human monocyte-derived dendritic cells (MoDC) were unable to up-regulate the co-stimulatory molecules CD80 and CD86 required for T cell activation. Exogenous TLR3 stimulation did not overcome the IAV-mediated inhibition of co-stimulatory molecule expression in MoDC. However, TLR3 stimulation using PolyI:C led to an augmented pro-inflammatory cytokine response. We reveal that ssON effectively inhibited PolyI:C-mediated pro-inflammatory cytokine production in MoDC, notably, ssON treatment maintained an interferon response induced by IAV infection. Accordingly, RNAseq analyses revealed robust up-regulation of interferon-stimulated genes in IAV cultures treated with ssON. We next measured reduced IAV production in MoDC treated with ssON and found a length requirement for its anti-viral activity, which overlapped with its capacity to inhibit uptake of PolyI:C. Hence, in cases wherein an overreacting TLR3 activation contributes to IAV pathogenesis, ssON can reduce this signaling pathway. Furthermore, concomitant treatment with ssON and IAV infection in mice resulted in maintained weight and reduced viral load in the lungs. Therefore, extracellular ssON provides a mechanism for immune regulation of TLR3-mediated responses and suppression of IAV infection *in vitro* and *in vivo* in mice.

## Introduction

According to new estimates by the United States Centers for Disease Control and Prevention (US-CDC), the World Health Organization and global health partners (www.who.int), seasonal influenza infections annually cause up to 650.000 deaths worldwide. The annual influenza vaccines incorporate variants of influenza A(H1N1), A(H3N2), B/Yamagata and B/Victoria, which WHO predicts will dominate the following season. However, the match between predicted and circulating strains can vary, leading to variable efficacy of the vaccine. In addition, influenza viruses pose a constant threat of human pandemics due to the risk of transmission of new variants from animals for which we currently lack appropriate vaccines (1, 2). There is therefore a pressing need to develop treatments targeting key steps in the life cycle utilized by many influenza viruses, thereby increasing the likelihood to achieve broad anti-viral activity (3). The pivotal step of viral entry into cells, may be an Achilles' heel accessible for novel broad antiviral compounds as it is shared by many viruses after binding to cellular receptors. We recently reported that certain ssON's have the capacity to inhibit both clathrin mediated and caveolin dependent endocytosis (4). Due to the notion that IAV utilizes cellular endocytic machinery for viral entry, we here assessed ssON's capacity to affect IAV infection.

Influenza strains infect respiratory epithelial cells, which may lead to cytopathic viral effects (5). Attachment to cells via sialic acid receptors enables endocytic uptake resulting in recognition of the virus via pattern recognition receptors (PRRs). PRRs trigger cytokine responses and induction of protective immunity, but they might also contribute to immune pathology (5). Chemokines released from the epithelial cells recruit neutrophils and mononuclear phagocytes including dendritic cells (DCs), leading to augmented cytokine, and interferon (IFN) responses (6). The IFN and JAK-STAT signaling pathways induce hundreds of interferon stimulated genes (ISGs) that are effectors able to limit viral replication (7). Depending on the influenza strain, there will be a differential strength of the immune pathological consequences (5).

Similarly to epithelial cells, DCs detect specific components of pathogens and endogenous factors released during cellular stress or from dying cells through their PRRs (8). Triggering of PRRs in MoDCs normally leads to up-regulation of the co-stimulatory molecules CD80 and CD86, which are required for effective priming of naïve T cells, as well as production of polarizing cytokines (9). However, immune protection by DCs can be compromised during IAV infection if they get infected, resulting in aberrant cytokine responses, and altered interactions with other immune cells (10). Pathogenic infections and tissue damage cause the release of nucleic acids, which also activate PRRs, leading to type I IFN, and pro-inflammatory cytokine production (8). The nucleic acid sensing PRRs include RIG-I like receptors (RIG-I, LGP2, DDX3, and MDA5), cytosolic DNA sensors, and a subgroup of TLRs consisting of TLR3, 7, 8, and 9, as well as murine TLR13 (8, 11). Influenza infections are recognized by TLRs, such as TLR7 that binds ssRNA and TLR3, which senses dsRNA in the endosomes (6, 12). TLR3 activation is mediated by TRIF, which leads to activation of both NFκB and IRF3. The source of TLR3 ligands generated during influenza infection remains to be determined as dsRNA is not generated during influenza virus replication due to the action of RNA helicase DDX39B (13). However, cellular stress and cell death also cause dsRNA release contributing to inflammation (6), which may explain TLR3 activation during influenza A infection. The negative impact of TLR3 activation during influenza was revealed using TLR3^−/−^ mice, which survived longer than wild-type mice following lethal influenza infection despite sustained high viral load in the lungs (14). TLR3^−/−^ mice displayed reduced chemokine expression in the lungs, fewer infiltrating leukocytes, and CD8^+^ T cells after lethal challenge. Thus, although TLR3 induces ISGs that can restrict viral replication, it simultaneously promotes recruitment of immune cells into the lungs that cause damage to the host.

Several influenza strains were previously shown to inhibit induction of co-stimulatory molecules in MoDC (15), while others reported induction of their expression but an impaired capacity to cross-present antigens (16). We sought to evaluate whether the pandemic H1N1 A/Cal/07/2009 blocked up-regulation of CD80 and CD86 and if so, whether TLR3 stimulation using PolyI:C), could overcome the inhibition. The expression of co-stimulatory molecules was measured by flow cytometry of both infected and non-infected MoDC using an antibody specific for viral nucleoprotein (NP). To further evaluate the impact of TLR3 on early innate responses upon IAV infection, we performed transcriptomic analyses and measured both cytokine and IFN-responses in IAV infected MoDC cultures after stimulation with PolyI:C or an immunomodulatory ssON with the capacity to inhibit TLR3 activation (4).

We recently found that certain ssONs inhibit endocytic pathways used by cargo destined for TLR3/4 signaling endosomes in MoDC (4). Both single-stranded DNA and RNA conferred the endocytic inhibition, which was concentration dependent and required a certain ssON length. The ssON-mediated inhibition modulated signaling downstream of TLRs that localized within the affected endosomal pathway (4). We, furthermore, demonstrated that ssON inhibits clathrin mediated endocytosis (CME) (4), which is a cellular entry pathway utilized by influenza A viruses (17–19). Therefore, we here also investigated whether ssON could affect IAV infection *in vitro* in human cells and in a murine *in vivo* challenge model.

## Materials and Methods

### IAV Infection and Reagents

Stock of pandemic H1N1 virus strain A/Cal/07/2009 was kindly provided by Bertin-Pharma, France. MoDC were mock-exposed or exposed to IAV or heat inactivated IAV (HI IAV, 30 min at 56°C) at a multiplicity of infection (MOI) of 0.02, 0.2, 1, or 2 for 4 h at 37°C 5%CO_2_ in serum-free RPMI medium, washed in pre-warmed complete RPMI medium and distributed in 24 wells plates (0.5 × 10^6^/mL). Cells were then treated or not with the following molecules: synthetic, endotoxin-free, completely phosphorothioate-modified oligonucleotides named ssON (0.5 μM; Integrated DNA Technologies), or an oligonucleotide with the naturally occurring phosphodiester backbone (ssON PO), high molecular weight PolyI:C (25 μg/mL; InvivoGen) or the combination of both, referred as ssON/PolyI:C. The sequence of 35 bases long ssON is: 5′-G^*^A^*^A^*^G^*^T^*^T^*^T^*^T^*^G^*^A^*^G^*^G^*^T^*^T^*^T^*^T^*^G^*^A^*^A^*^G^*^T^*^T^*^G^*^T^*^T^*^G^*^G^*^T^*^G^*^G^*^T^*^G^*^G^*^T^*^G-3′, the sequence of the 30-mer is: 5′-A^*^G^*^T^*^T^*^T^*^T^*^G^*^A^*^G^*^G^*^T^*^T^*^T^*^T^*^G^*^A^*^A^*^G^*^T^*^T^*^G^*^T^*^T^*^G^*^G^*^T^*^G^*^G^*^T^*^G-3′, the 25-mer: 5′-T^*^T^*^T^*^G^*^A^*^G^*^G^*^T^*^T^*^T^*^T^*^G^*^A^*^A^*^G^*^T^*^T^*^G^*^T^*^T^*^G^*^G^*^T^*^G^*^G-3′, the 20-mer: 5′-T^*^G^*^A^*^G^*^G^*^T^*^T^*^T^*^T^*^G^*^A^*^A^*^G^*^T^*^T^*^A^*^T^*^T^*^G^*^G-3′ and the 15-mer: 5′- G^*^G^*^T^*^T^*^T^*^T^*^G^*^A^*^A^*^G^*^T^*^T^*^G^*^T^*^T-3′, wherein the phosphorothioate modifications are indicated by ^*^.

### MoDC Culture and Flow Cytometry

Monocytes were isolated from buffy coats using Ficoll centrifugation (Lymphoprep; Axis Shield) after negative selection using the RosetteSep Monocyte Enrichment Kit (StemCell Technologies). Monocyte-derived DC (MoDC) were obtained after 6 days of differentiation in complete RPMI medium (RPMI 1,640, 1 mM sodium pyruvate, 10 mM HEPES, 2 mM L-glutamine, 1% Penicillin/Streptomycin, Hyclone GE Healthcare, and 10% FBS, Sigma) complemented with GM-CSF (250 ng/mL; PeproTech) and rIL-4 (6.5 ng/mL; R&D Systems). Cells were seeded at a density of 5 × 10^5^ cells/mL and after 3 days of differentiation, 50% of the medium was replaced and new cytokines added. Staining for flow cytometry was done before and at indicated time points post viral infection. MoDC were incubated with LIVE/DEAD® Fixable near-IR Dead Cell Stain Kit (Life Technologies) followed by staining with CD14-PE-Cy7 (MφP9), CD1a-BV510 (HI149), CD80-PE (L307.4), and CD86-APC (2331 FUN-1) from BD Biosciences. The mouse anti-IAV NP mAb (H16-L10-4R5; Merck Millipore) was detected with a secondary Ab coupled to Alexa Fluor 488 fluorochrome with the Zenon® Kit (Invitrogen). Acquisition was done on a Fortessa flow cytometer (BD Biosciences) and analysis was performed with FlowJo software (Tree Star, version 10.2).

### Uptake Studies in MoDC

MoDC were exposed to PolyI:C-Alexa488, with or without addition of ssON, on ice in complete 10% RPMI media (or serum free media for PO ON uptake studies), and then transferred to 37°C for 45 min, as previously described (4). Cells were washed with cold PBS and fixed (Cytofix, BD Bioscience) before monitoring of the fluorescent signal by flow cytometry (Fortessa, BD Biosciences). Data were analyzed with FlowJo software (Tree Star, version 9.6.4). For microscopy, MoDC were adhered to poly-L-lysine coated glass slides for 2–4 h. Cells were treated with Poly I:C-Cy3 (orange color) at 37°C or 4°C for 45 min in the presence or absence of 0.5 μM unlabeled ssON 35 PS. Cells were washed with PBS and stained with wheat germ agglutinin-Alexa633 (Invitrogen) for 10 min prior to fixation in 3.7% Formaldehyde (Sigma). Images were acquired in a LSM800 airy scan confocal microscope (Zeiss) using the 63X oil lens and the images were analyzed using the Zen blue software (Zeiss).

### RNA Extraction and Real-Time PCR

At 24 or 48 h post infection (p.i), supernatants or MoDC were harvested and kept at −80°C until RNA purification was performed. RNeasy® Plus Mini kit (QIAGEN) was used to extract cellular RNA while the QIAamp® Viral RNA mini kit (QIAGEN) was used for viral RNA purification from supernatants, following manufacturer' spin protocol. Viral load was determined by qRT-PCR (SuperScript® III Platinum® One-Step Quantitative RT-PCR System; Life Technologies) using pandemic H1N1 virus strain A/Cal/07/2009 as internal standard. The following primer sequences 5′-3′ specific for IAV Cal07/09 *HA* segment were used Forward: GGCTGCTTTGAATTTTACCACAA, Reverse: TTTGGGTAGTCATAAGTCCCATTTT and the probe sequence was *FAM-*TGCGATAACACGTGCATGGAAAGTGTC*-TAMRA* (20).

### RNA Sequencing and Differential Expression Analysis

Purified RNA molecules were submitted to the National Genomics Infrastructure Sweden Stockholm (NGI) for sequencing. The RNA sequencing was performed with the TruSeq RiboZero kit from Illumina, 25 M reads per sample and 2 × 125 bp. Fastq files were obtained from NGI and the read quality were assessed using FastQC (Version 0.11.5) Trim Galore (Version 0.3.6) was used for adapter removal and quality trimming with a quality threshold of 20 on the Phred scale. Obtained high-quality reads were mapped to Homo sapiens UCSC hg38 (GRCh38.77) reference genome using STAR aligner (version 2.5) with default values and the parameter out Reads Unmapped set to FastX in order to extract the unmapped reads. After STAR alignment, the count data for the aligned reads were generated with HTSeq-count (version 0.6.1). The-m parameter was set to union. Next, the count data sets were imported into the statistical software R (version 3.5.1). The R/Bioconductor package DESeq2 (version 1.22.1) was used to conduct differential expression (DE) analysis according to the workflow outlined in the vignette. Hierarchical clustering was performed on a sample-to-sample distance matrix that was calculated on regularized logarithm transformed counts with the *dist* function in R. In the DESeq2 pipeline, the design formulae was set to “~ donor + control” i.e., to analyse the effect of condition while controlling for the donor specific differences. For each comparison, filtering was performed prior to analysis by removing all genes that had zero counts across samples. Shrinkage of log_2_ fold changes was performed according to the DESeq2 pipeline. Genes with adjusted *p*-value below 0.05 after Benjamini-Hochberg adjustment, were labeled as differently expressed.

### Pathway Analysis

Ingenuity Pathway Analysis (IPA) software (Build version 486068M, Content version 46901286 release date 20181121) (Ingenuity Systems) was used to identify canonical signaling pathways. To calculate significance of enrichment (Fisher's exact test, performed within the software), the reference molecule set was Ingenuity Knowledge Base (Genes only). Results tables were exported from R after DESeq2 and prior to IPA analysis filtered by adjusted *p*-value below 0.05.

### Cytokine/IFN Secretion

Supernatants were collected from cells at given time points post infection and secretion of cytokines/IFN was measured by standard ELISA according to manufacturer's instructions (IL-6, IL-12/23(p40), IL-12(p70), IL-29; Mabtech, IFN-α; PBL). Protein amount was monitored by 3,3′,5,5′-Tetramethylbenzidine (TMB) absorbance at 450 nm.

### Mouse Experiments

H1N1, A/England/195/2009 was isolated by Public Health England (UK) in SIAT-MDCK cells (21). Prior to use in mice, viruses were propagated in MDCK cells, in serum-free DMEM supplemented with 1 μg/ml trypsin. The virus was harvested 3 days after inoculation and stored at −80°C. Viral titer was determined by plaque assay as described previously (22). 6–10-week-old female BALB/c mice were obtained from Charles River UK Ltd. (Leeds, UK) and kept in specific-pathogen-free conditions in accordance with United Kingdom's Home Office guidelines. All work was approved by the Animal Welfare and Ethical Review Board (AWERB) at Imperial College London. Studies followed the ARRIVE guidelines. For infections, mice were anesthetized using isoflurane and infected intranasally (i.n.) with 4 × 10^4^ influenza virus or sterile PBS 100 μl.

Mice were culled using 100 μl intraperitoneal pentobarbitone (20 mg dose, Pentoject, Animalcare Ltd., UK) and tissues collected as previously described (23). Blood was collected from femoral veins and sera isolated after clotting by centrifugation. Lungs were removed and homogenized by passage through 100-μm cell strainers, then centrifuged at 200 × *g* for 5 min. Supernatants were removed and the cell pellet treated with red blood cell lysis buffer (ACK; 0.15 M ammonium chloride, 1 M potassium hydrogen carbonate, and 0.01 mM EDTA, pH 7.2) before centrifugation at 200 × *g* for 5 min. The remaining cells were resuspended in RPMI 1,640 medium with 10% fetal calf serum, and viable cell numbers determined by trypan blue exclusion. Viral load *in vivo* was assessed by Trizol extraction of RNA from frozen lung tissue disrupted in a TissueLyzer (Qiagen, Manchester, UK). RNA was converted into cDNA and quantitative RT-PCR was carried out using bulk viral RNA, for the influenza M gene and mRNA using 0.1 μM forward primer (5′-AAGACAAGACCAATYCTGTCACCTCT-3′), 0.1 μM reverse primer (5′-TCTACGYTGCAGTCCYCGCT-3′), and 0.2 μM probe (5′-FAM-TYACGCTCACCGTGCCCAGTG-TAMRA-3′) on a Stratagene Mx3005p (Agilent technologies, Santa Clara, CA, USA). M-specific RNA copy number was determined using an influenza M gene standard plasmid (22).

### Statistical Analysis

All data were analyzed using Prism software (GraphPad, version 6.07). Unless stated otherwise, the non-parametric Kruskal-Wallis unpaired test followed by Dunn's post-test was used to compare against the control (represented with a line), while different treatment groups were compared using non-parametric Mann-Whitney unpaired test (represented with a bar). ns: not significant, ^*^P < 0.05, ^**^P < 0.01, ^***^P < 0.001.

## Results

### Limited Cell Death in MoDC Infected With Pandemic H1N1 A/Cal/07/2009

**Figure 1:**
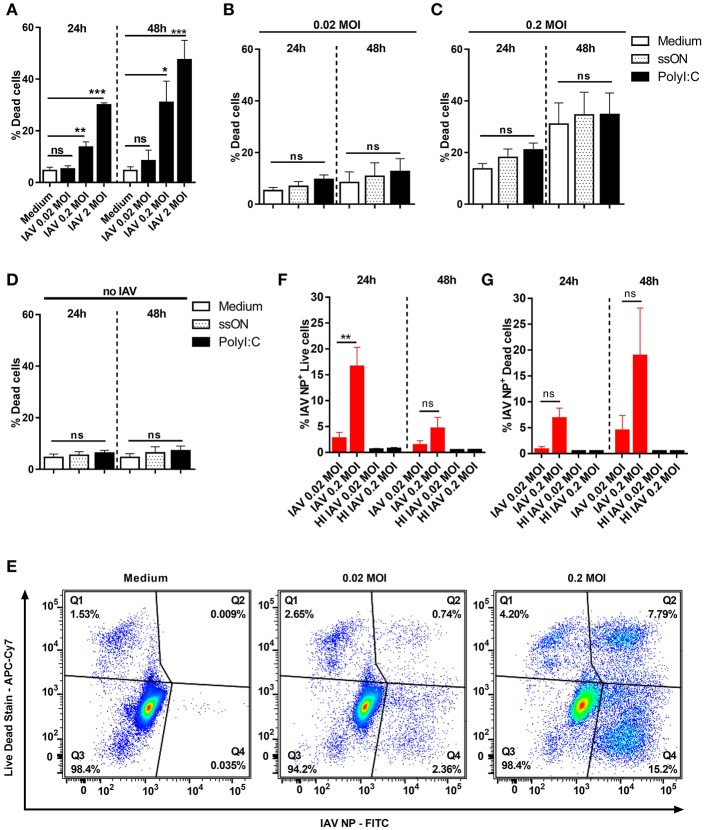
Cell death in IAV-infected and non-infected MoDC. MoDC were mock or IAV-infected at indicated MOI for 4 h and the viability was measured by flow cytometry after culture in medium or stimulation with ssON or PolyI:C. **(A–D)** The frequency of dead MoDC was measured 24 and 48 h p.i. by flow cytometry. Graphs represent mean ± SEM with *n* = 8–12 (24 h) and *n* = 5–8 (48 h) except for MOI of 2 (*n* = 2 in triplicates for both time points). In **(A)**, statistical comparisons were made between mock infection (“Medium”) and IAV infections. In **(B–D)**, the different treatments with ligands were compared with IAV infection without any ligands (white bars). Statistics were calculated using Kruskal-Wallis one-way ANOVA test with Dunns multiple comparisons test and alpha set to 0.05. **(E)** Representative flow cytometry plots showing the gating strategy to determine dead non-infected (Q1), dead IAV-infected NP^+^ (Q2), live non-infected (Q3), and live IAV^−^infected NP^+^ (Q4) MoDC. **(F,G)** Bar graphs showing percentages of live **(F)** and dead **(G)** IAV-infected NP^+^ MoDC over time (red bars). Mean ±SEM with *n* = 9 for 24 h and *n* = 5 for 48 h except for the control HI IAV (HI 0.02, *n* = 3 and 1, HI 0.2, *n* = 4 and 2, 24, and 48 h respectively). *P*-value: not significant (ns) *P* > 0.05; *P ≤ 0.05; **P ≤ 0.01; ***P ≤ 0.001.

We here sought to study the role of TLR3-signaling during pandemic influenza infection by either adding a TLR3 agonist, or inhibiting the response by ssON. Therefore, we first monitored cell death following *in vitro* IAV infection, at different time points, as we wanted to avoid release of dsRNA due to cell death, which may affect innate immune responses (6). We used flow cytometry to quantify cell death among MoDC infected with the pandemic H1N1 A/Cal/07/2009 strain (Figure 1A). The lowest MOI of 0.02 did not induce any significant cell death either 24 h (<5%) or 48 h (<10%) post infection (p.i.). However, MOI of 2 induced significant cell death with ~30 and 50% dead cells 24 and 48 h p.i., respectively. Intermediate MOI 0.2 resulted in intermediate cell death, suggesting a dose-dependent response. Addition of the TLR3 agonist PolyI:C or the inhibitor ssON in pre-titrated concentrations 25 μg/ml and 0.5 μM, respectively (21), did not significantly affect MoDC death in IAV infected cultures (Figures 1B,C). Control experiments, without IAV infection, showed that PolyI:C and ssON alone did not induce cell death (Figure 1D).

To investigate whether dying MoDC were infected with IAV, we performed intracellular staining using an anti-IAV NP mAb. As shown in Figure 1E, such staining allowed for discrimination between live IAV NP^+^ and dead IAV NP^+^ MoDC as well as live and dead non-infected bystander MoDC. As expected, based on the viability results, we predominantly found IAV NP staining in live MoDC at 24 h p.i. (Figure 1F), while there was an accumulation of IAV NP^+^ cells among dead cells 48 h p.i. (Figure 1G). The intracellular NP staining revealed that the frequency of IAV NP^+^ live cells 24 h p.i. using 0.02 MOI was relatively low (2.8% ± 1.1), but increased to 16.7% ± 3.7 with 0.2 MOI. MoDC incubated with heat-inactivated (HI) IAV did not show any detectable NP^+^ cells or any cell death 24 h or 48 h post-exposure, as expected (Supplementary Figure 1). We did not detect any IAV-NP signal in MoDC analyzed 2 h p.i. (data not shown) suggesting that the flow cytometry staining did not measure uptake of IAV.

These findings showed that the pandemic H1N1 A/Cal/07/2009 can infect MoDC without causing cell death, confirming the study by Hartmann et al. (24). However, when extending the kinetics and using the highest 2 MOI, we monitored viral inoculum dose-dependent induction of cell death in MoDC, which increased over time (Figure 1A). We therefore used 0.02 MOI and the intermediate 0.2 MOI, for the subsequent analyses as virus-induced cell death can *per se* influence the local milieu and immune responses.

### Inhibited Expression of Co-stimulatory Molecules in Pandemic H1N1 A/Cal/07/2009 NP^+^, but Not in Neighboring Non-infected MoDC

**Figure 2:**
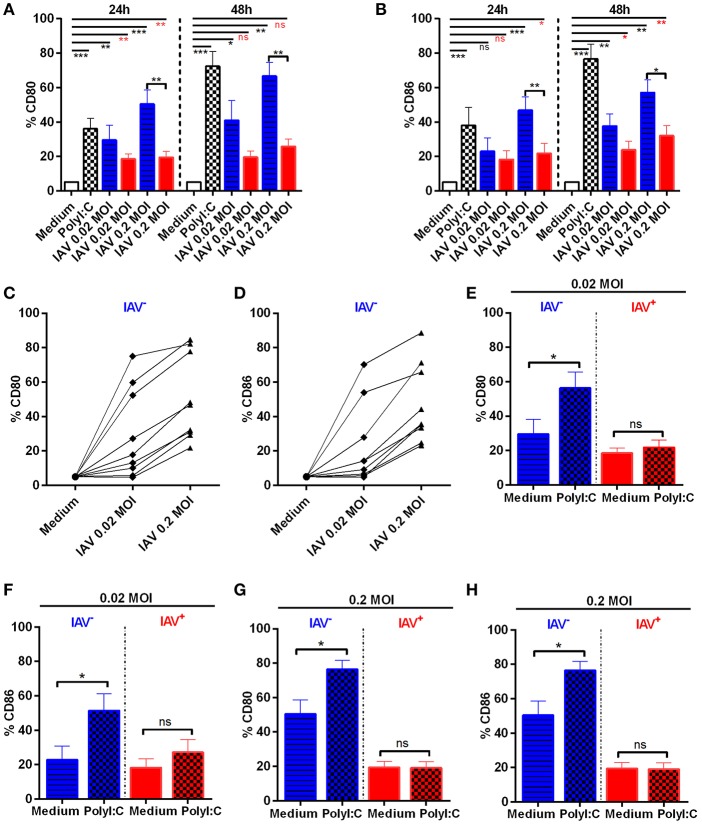
Expression of CD80 and CD86 in IAV-infected and non-infected MoDC. MoDC were mock or IAV-infected at indicated MOI for 4 h Statistical analysis were performed in comparison with mock infection (Medium) using one-way ANOVA with Dunn's multiple comparison test (alpha 0.05) and indicated with a line. Calculations made between two groups using two-tailed Mann-Whitney test were depicted with a bar. *P*-value: not significant (ns) *P* > 0.05; *P ≤ 0.05; **P ≤ 0.01; ***P ≤ 0.001. Frequencies of CD80 **(A)** and CD86 **(B)** expressing cells were measured by flow cytometry after gating on live non-infected (blue striped bar) and live IAV-infected NP^+^ (red bar) MoDC kept in medium alone after IAV infection (gating shown in Figure 1E and Supplementary Figure 2). Mean ± SEM with *n* = 8 (24 h) and *n* = 5 (48 h). Graphs representing CD80 **(C)** and CD86 **(D)** expression in live non-infected MoDC 24 h post mock or viral infection, at indicated MOI, for each donor (*n* = 9). MoDC were IAV-infected followed by addition of PolyI:C (chess bars) or kept in Medium alone. Graphs showing CD80 **(E,G)** and CD86 **(F,H)** expression on gated live non-infected (blue striped bar Medium) (blue chess bar PolyI:C) or live IAV-infected NP+ (red bar Medium) (red chess bar PolyI:C) MoDC exposed to IAV at 0.02 MOI **(E,F)** or 0.2 MOI **(G,H)** and indicated treatment 24 h p.i. Control cultures without PolyI:C are indicated as medium. Error bars show mean ± SEM with *n* = 8.

Several influenza strains, including pandemic H1N1, have previously been shown to impair the maturation of MoDCs (15). However, these studies did not always discriminate between infected and non-infected MoDCs in their analyses. Hence, to investigate the effect of H1N1 A/Cal/07/2009 infection on MoDC maturation, we specifically studied both infected and non-infected cells from the same cultures with the use of intracellular IAV NP staining. We found significant differences between IAV-infected and non-infected MoDC in their ability to up-regulate surface CD80 and CD86 (Figure 2); (example stainings and MFI in Supplementary Figure 2). Moreover, we detected a dose-dependent up-regulation of CD80/86 in IAV exposed MoDC, which were NP^−^. The susceptibility to MoDC activation in bystander non-infected cells were highly donor dependent (Figures 2A–D). Notably, the expression of co-stimulatory molecules was significantly lower in the IAV^+^ MoDC as compared with the IAV^−^ MoDC analyzed from the same donors (Figures 2A,B). For comparison, MoDCs were treated with PolyI:C, which as expected, up-regulated both co-stimulatory molecules at 24 and 48 h after infection compared to unstimulated MoDC (Figures 2A,B). The expression of co-stimulatory molecules increased over time in the IAV exposed NP^−^ MoDC and in the PolyI:C-stimulated MoDCs, but it did not appear to increase over time in the IAV^+^ MoDC (Figures 2A,B). These data show an impaired maturation in IAV^+^ MoDCs, while neighboring non-infected (NP^−^) MoDCs were able to up-regulate the surface expression of both CD80 and CD86.

We next assessed whether stimulation with PolyI:C could augment the expression of CD80 and CD86 in MoDC cultures exposed to IAV (Figures 2E–H). Accordingly, the IAV infection culture condition induced CD80/86 surface expression *per se* in the neighboring non-infected (NP-) MoDCs (Figures 2C,D), which was significantly further up-regulated by PolyI:C treatment (Figures 2E–H blue bars). However, PolyI:C treatment did neither induce CD80 nor CD86 expression in the IAV^+^ MoDCs (Figures 2E–H red bars). Stimulation with ssON did not affect expression of CD80 or CD86 in neither IAV^−^ nor IAV^+^ MoDC (Supplementary Figure 3). However, the TLR3 inhibitor ssON reduced CD80 and CD86 expression in the presence of PolyI:C stimulation (Supplementary Figure 3). Altogether, these data demonstrate that IAV^+^ MoDC were refractory to PolyI:C-mediated induction of maturation. Furthermore, neighboring IAV^−^ MoDC responded to PolyI:C treatment.

### IAV MoDC Cultures Displayed Clear Up-Regulation of ISGs but Limited Effect on Inflammasome Genes

**Figure 3:**
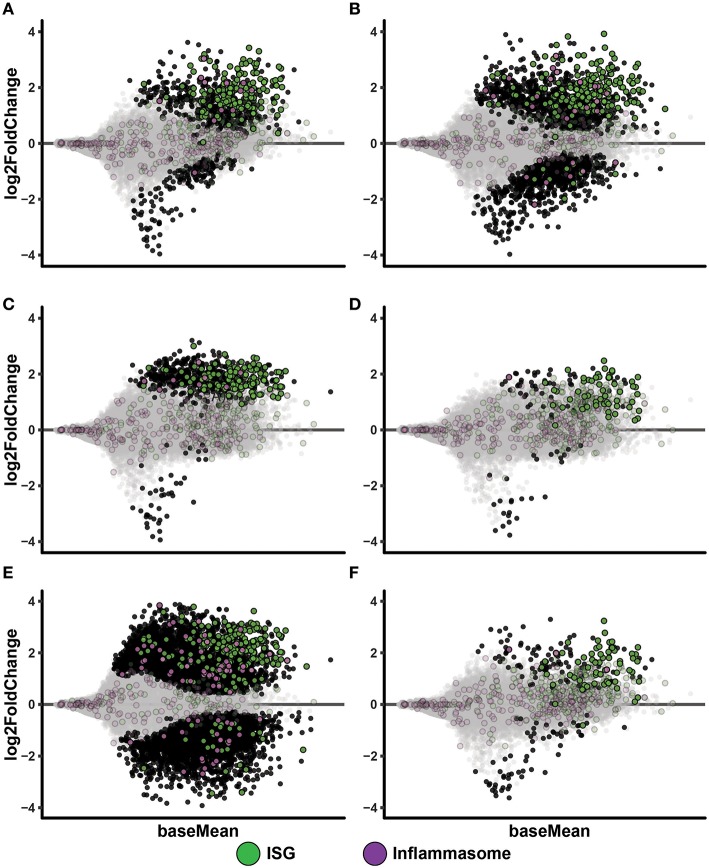
IAV infected MoDC display up-regulation of ISGs. MA-plots after RNA sequencing of MoDC samples. Each comparison includes a total of four samples. Genes with adjusted p-value below 0.05 are fully colored (ISG, green; inflammasome, purple; rest, black). **(A)** Medium control vs. IAV 0.02 MOI. **(B)** Medium control vs. IAV 0.2 MOI. **(C)** Medium control vs. PolyI:C stimulation. **(D)** Medium control vs. ssON. **(E)** Medium control vs. IAV 0.2 MOI with PolyI:C stimulation. **(F)** Medium control vs. IAV 0.2 MOI with ssON stimulation.

To get further mechanistic unbiased insights to events occurring in these culture conditions, we conducted RNAseq analyses. Hierarchical clustering of samples display sample treatment condition as the major driver of sample similarity, although some donor specific differences are present (Supplementary Figure 4) and accounted for in the subsequent DEA (Materials and Methods RNA sequencing and differential expression analysis). MA plots of IAV infected MoDC cultures displayed a clearly diverged transcriptomic profile compared to non-infected MoDC (Figures 3A,B). A large proportion of the up-regulated genes during IAV infection belonged to the ISG category according to list published by Barouch et al. (25) (Supplementary Table 1) and included factors with anti-flu activity (Supplementary Table 2) [generated by using (7) and data shown in Supplementary Figures 5A,B]. We noted that IAV infection led to increased TLR3 expression in MoDC, while ssON treatment of IAV infected samples prevented this up-regulation (Supplementary Table 3). However, IAV did not induce extensive inflammasome gene signatures as compared to those reported for SIV using the same gene lists for analyses (Supplementary Table 4) (25), which is in accordance with pandemic IAVs capacities to inhibit activation of the NLRP3 inflammasome (26). However, further analyses is required to establish the relative contribution of NLRP3 signaling during IAV infections, as recently reviewed in Tate and Mansell (27). The PolyI:C stimulated cultures showed a similar gene expression pattern, with an up-regulation of ISGs including up-regulation of anti-flu factors and limited up-regulation of inflammasome gene signatures (Figure 3C and Supplementary Figure 5C). Non-infected MoDC stimulated with ssON did not display robust induction of transcription (Figure 3D), in agreement with our previous report (4).

IPA Pathway analyses of PolyI:C activated MoDC revealed robust up-regulation of “Interferon Signaling,” and also showed significant up-regulation of genes (positive Z-score) in “Activation of IRF by Cytosolic Pattern Recognition Receptors” and “Dendritic Cell Maturation,” which are expected after triggering of innate immune responses (Table 1). Furthermore, RNAseq analyses of MoDC infected with IAV, similarly, showed a dominant activation of “Interferon Signaling” with up-regulation of the majority of genes annotated to this pathway (Table 1). Additional innate immune pathways revealed upon IAV infection of the MoDC were “Crosstalk between Dendritic Cells and Natural Killer Cells” and “Caveolar-mediated Endocytosis Signaling” (Table 1). There were no significant pathways revealed in MoDC cultures treated with ssON alone, in accordance with the MA plots (Figure 3D and Supplementary Figure 5D).

**Table 1:** PA analyses of RNA sequencing of MoDC.

**Medium vs. Polyl:C**	
**Pathway**	**log_10_(*p*-value)**
Interferon signaling	19.4
Crosstalk between dendritic cells and natural killer cells	11.4
Type I diabetes mellitus signaling	10.6
Activation of IRF by cytosolic pattern recognition receptors	9.56
Dendritic cell maturation	7.12
Th1 and Th2 activation pathway	6.76
Th1 pathway	5.88
Role of pattern recognition receptors in recognition of bacteria and viruses	5.80
**Medium vs. IAV 0.2 MOI**	
Interferon signaling,	17.2
T cell exhaustion signaling pathway	12.6
Crosstalk between dendritic cells and natural killer cells	9.98
Cytotoxic T lymphocyte-mediated apoptosis of target cells	7.78
Caveolar-mediated endocytosis signaling	7.69
Phospholipase C signaling	7.63
Th1 pathway	7.31
Retinoic acid mediated apoptosis signaling	7.30
**Medium vs. IAV 0.2 MOI + Polyl:C**	
Mitochondrial dysfunction	25.7
EIF2 signaling	21.9
Oxidative phosphorylation	19.5
T cell exhaustion signaling pathway	16.1
Th1 and Th2 activation pathway	15.2
Th1 pathway	14.1
Crosstalk between dendritic cells and natural killer cells	13.7
Activation of IRF by cytosolic pattern recognition receptors	13.6
**Medium vs. 0.2 MOI + ssON**	
Interferon signaling	17.9
Activation of IRF by cytosolic pattern recognition receptors	8.58
Role of pattern recognition receptors in recognition of bacteria and viruses	7.60
Role of lipids/lipid rafts in the pathogenesis of influenza	7.55
Superpathway of cholesterol biosynthesis	7.00
Role of hypercytokinemia/hyperchemokinemia in the pathogenesis of influenza	5.83
Role of RIG1-like receptors in antiviral innate immunity	5.77
Retinoic acid mediated apoptosis signaling	4.89

To get unbiased analyses of transcriptional changes occurring upon TLR3 activation during IAV infection of MoDC, MA plots were generated displaying some of the significantly up- and down-regulated genes in the presence of either PolyI:C or ssON (Figures 3E,F). The IAV MoDC cultures displayed a strong differential ISG expression after treatment with PolyI:C (Figure 3E) but with negligible effect on anti-flu genes (Supplementary Figure 5E). Hence, there seems to be no additive effect when treating already IAV infected MoDC cultures with PolyI:C in terms of up-regulation of anti-flu genes. The IPA analyses revealed a negative z-score for “EIF2 Signaling” and “Oxidative Phosphorylation” as well as a positive z-score for “Activation of IRF by Cytosolic Pattern Recognition Receptors” but also a highly significant score for mitochondrial dysfunction (Table 1).

The IAV cultures treated with ssON revealed an up-regulation of ISGs (Figure 3F), including anti-flu genes (Supplementary Figure 5F). IPA analyses comparing cultures kept in medium vs. IAV/ssON (wherein ssON was added 2 h post-infection), revealed a strong up-regulation of “Interferon Signaling” and other innate pathways associated with IAV infection such as “Activation of IRF by cytosolic Pattern Recognition Receptors,” “Role of Pattern Recognition Receptors in Recognition of Bacteria and Viruses” (Table 1). Altogether, this suggests that ssON does not completely block innate signaling pathways triggered upon IAV infection and that substantial IFN-mediated effects prevail. The IPA analyses furthermore revealed a significant downregulation (negative z-score) of “Superpathway of Cholesterol Biosynthesis” in the IVA infected cultures treated with ssON, which is intriguing in relation to previous reports showing that long non-coding RNA can inhibit cholesterol biosynthesis (28).

**Figure 4:**
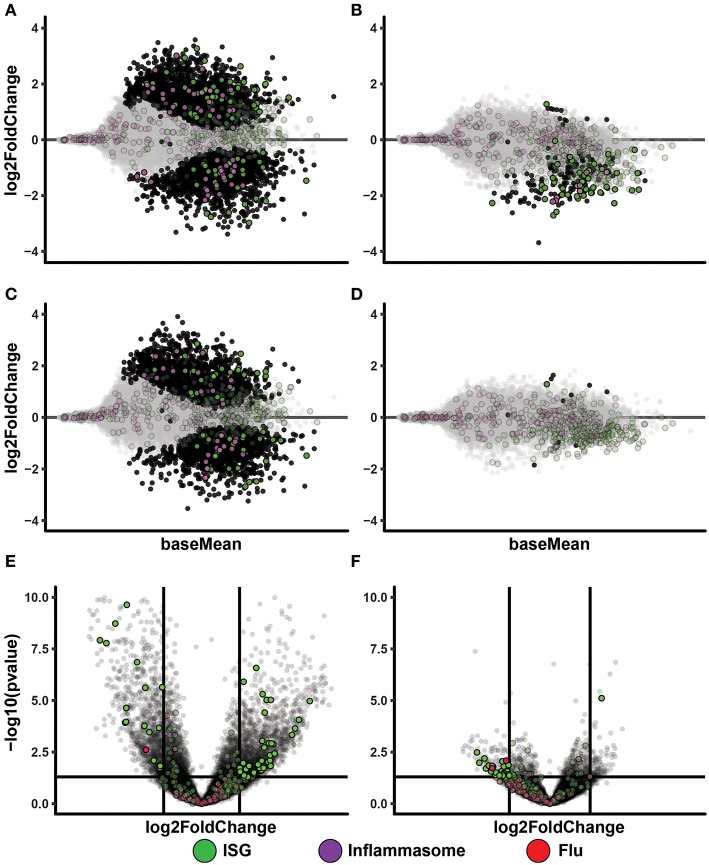
Transcriptional changes after TLR3 activation of IAV infected MoDC. **(A–D)**: MA-plots after RNA sequencing of MoDC samples. Each comparison includes a total of four samples. Genes with adjusted *p*-value below 0.05 are fully colored (ISG = green, inflammasome = purple, rest = black). **(A)** IAV 0.02 MOI vs. IAV 0.02 MOI/PolyI:C. **(B)** IAV 0.02 MOI vs. IAV 0.02 MOI/ssON. **(C)** IAV 0.2 MOI vs. IAV 0.2 MOI/PolyI:C. **(D)** IAV 0.2 MOI vs. IAV 0.2 MOI/ssON. **(E,F)**: Volcano-plots after RNA sequencing of MoDC samples. Each comparison includes a total of four samples. The horizontal line is drawn at -log10(*p*-value) = 0.05 and the vertical lines are drawn at absolute log2foldchange = 1. Genes included in the ISG and flu lists and that pass these thresholds are fully colored (ISG = green, flu = red). **(E)** IAV 0.2 MOI vs. IAV 0.2 MOI with PolyI:C stimulation. **(F)** IAV 0.2 MOI vs. IAV 0.2 MOI with ssON stimulation.

To get insight to the differential gene expression occurring in the PolyI:C treated IAV cultures, we made MA-plots and volcano plots comparing IAV infected cultures vs. IAV infected cultures treated with PolyI:C (IAV 0.02 Figure 4A, IAV 0.2 Figures 4C,E). It is clear that these cells responded to PolyI:C with extensive both up- and down-regulation of thousands of genes, some belonging to the ISG and inflammasome gene categories. We performed the same comparisons in cultures treated with ssON (IAV 0.02 Figure 4B, IAV 0.2 Figures 4D,F). However, the effect on gene expression in IAV infected cultures treated with ssON was again modest.

Altogether, these data showed induction of IFN-signaling pathways in the IAV cultures regardless of whether cells were treated with PolyI:C or ssON. These data also demonstrated that IAV-infected cultures treated with PolyI:C resulted in further up-regulation of many genes, while ssON treatment did not have any major effect on the baseline gene expression induced by the IAV infection.

### ssON Limits dsRNA-Mediated Top-Up of Pro-Inflammatory Cytokines and IFNs in IAV Cultures

**Figure 5:**
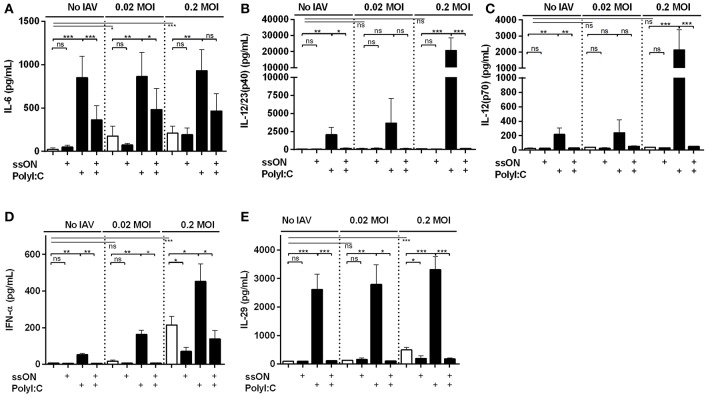
Cytokines and IFN secretion in IAV infected MoDC cultures. Culture supernatants were collected 24 h p.i and subjected to ELISA. Statistical analyses performed in comparison with mock infection (Medium) are indicated with a line and calculations made between two groups are depicted with a bar. Mean ±SEM. **(A)** IL-6 data were from at least seven independent donors. **(B)** IL-12/23(p40), **(C)** IL-12(p70), **(D)** IFN-α, and **(E)** IL-29 are representative data from at least seven independent donors for the IAV 0.2 MOI-infected and non-infected cultures and from at least three donors analyzed independently for the 0.02 MOI cultures. *P*-value: not significant (ns) *P* > 0.05; *P ≤ 0.05; **P ≤ 0.01; ***P ≤ 0.001.

TLR3 activation results in expression of both pro-inflammatory cytokines and IFNs (9). To further validate some of the transcriptomic data, we collected supernatants from the IAV MoDC cultures with or without adding ligands that either activates (PolyI:C) or inhibits (ssON) TLR3 activation and measured cytokine contents by ELISA. We detected low amounts of IL-6 after IAV infection, which were significantly increased after treatment with PolyI:C (Figure 5A), confirming the results from the transcriptomics. Furthermore, we found that ssON treatment did not inhibit IL-6 production induced by the IAV culture condition *per se*, but significantly inhibited the augmented effect by PolyI:C (Figure 5A).

Additional cytokines produced by MoDC are the IL12A and IL12B family proteins. IAV did not *per se* provoke significant IL-12/23(p40) or IL-12(p70) secretions (Figures 5B,C). However, PolyI:C treatment induced secretion of both IL12/23 p40 and IL-12 p70, which was amplified by IAV infection. We observed that ssON completely inhibited PolyI:C-induced IL-12/23(p40) and IL-12 p70 secretions both with and without IAV infection (Figures 5B,C).

Finally, we analyzed a type I IFN-α and type III IL-29 secretions in these cultures as these anti-viral immune defenses induce ISGs (Figures 5D,E) (29). In the non-infected and 0.02 MOI infected cultures, PolyI:C induced significant IFN-α and IL-29 secretions, which were completely blocked by ssON. The 0.2 MOI IAV infection induced extensive IFN-α and IL-29 production, which were not completely inhibited by ssON (Figures 5D,E). However, PolyI:C induced an elevated IFN-α and IL-29 production in the IAV infected cultures and this additional top-up was inhibited by ssON (Figures 5D,E).

Altogether, these data demonstrate PolyI:C-mediated induction of several pro-inflammatory cytokines and IFNs in the IAV cultures, confirming the transcriptomic analyses. Likewise, the data shows that ssON was able to effectively inhibit PolyI:C-mediated pro-inflammatory cytokine and IFN production but preserved cytokine and IFN responses induced by the IAV infection *per se*.

### Inhibition of Pandemic H1N1 in MoDC by ssON

**Figure 6:**
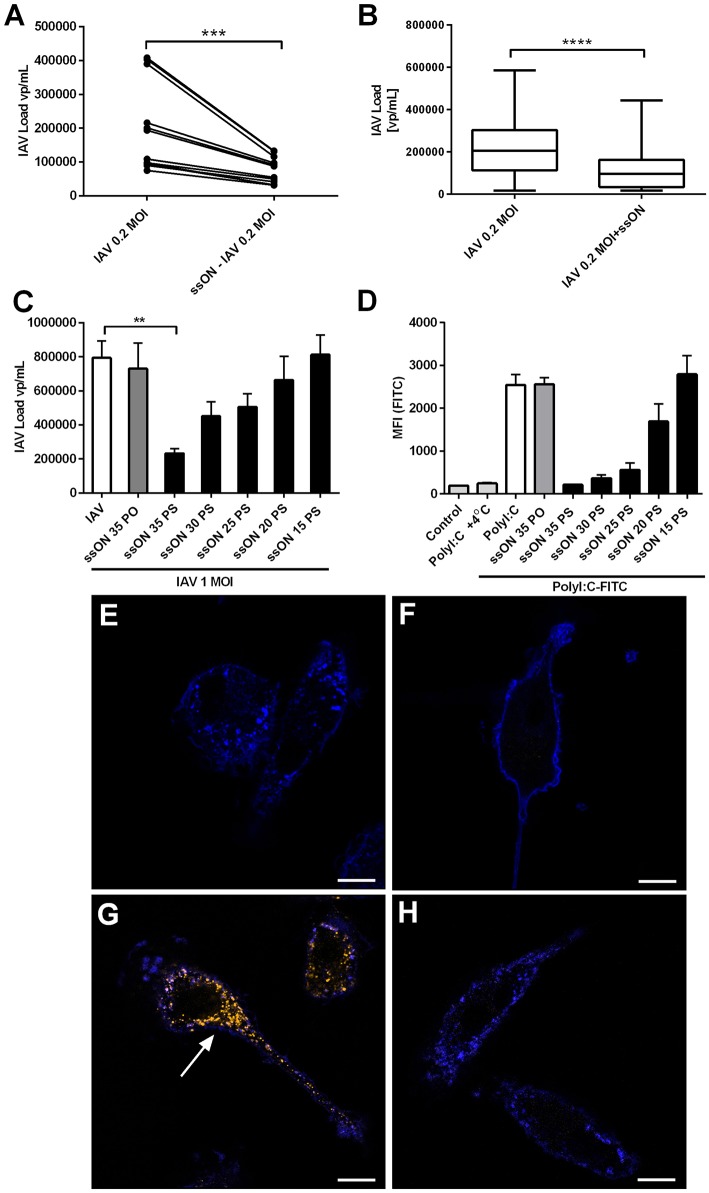
Decreased IAV content in supernatants of ssON treated MoDC cultures. **(A)** MoDC were treated with or without ssON during 2 h followed by IAV-infection at MOI of 0.2 for 2 h. IAV content was determined by qRT-PCR for the *HA* gene. Four individual donors were evaluated in triplicates. Wilcoxon paired two-tailed rank test was used ***P < 0.0005. **(B)** Treatment with ssON added 4 h after infection with IAV also showed a significant impact on the viral content detected in the supernatants. Data from 16 donors analyzed in triplicates. Wilcoxon paired two-tailed rank test was used ****P < 0.0001. **(C)** IAV content in supernatants at 24 h after infection with MOI of 1 in the absence (white bar) or presence of indicated ssON (sequences in M&M) with PO backbone or different lengths of PS ssON. Data from at least six samples **P ≤ 0.001. **(D)** MoDC were treated for 45 min at +37°C or +4°C with endocytic uptake markers PolyI:C-Alexa488 in the absence (white bar) or presence of indicated ssON with PO backbone or different lengths of PS ssON. Flow cytometry was used to quantify the fluorescent signal. Data from two donors run in duplicate. **(E–H)** Confocal microscopy of MoDC stained with a membrane marker, wheat germ agglutinin-Alexa633 (blue) and incubated for 45 min and kept **(E)** untreated, **(F)** with PolyI:C-Cy3 at 4°C, **(G)** PolyI:C-Cy3 at 37°C, and **(H)** PolyI:C-Cy3 at 37°C in the presence of ssON. Arrow points at uptake of PolyI:C-Cy3 (orange). Data representative of two donors. Scale bar 10 μM.

Extracellular ssON was recently shown to inhibit uptake of ligands taken up by CME and Caveolin-Dependent Endocytosis (CDE) (4), which are entry pathways used by influenza A (18). Therefore, we assessed if ssON has the capacity to influence IAV infection in MoDCs by measuring the amount of virus released into the supernatants. Initially and as expected, we found a viral inoculum dose-dependent induction of viral production. The viral inoculum dose of 0.02 MOI resulted in 62.998 vp/mL ± 12.513 and 0.2 MOI in 325.692 vp/mL ± 44.476 24 h p.i. (Supplementary Figure 6A). We found that pre-treatment with ssON for 2 h prior to addition of the virus and maintaining ssON in culture during the 24 h culture period, resulted in a significant reduction of the viral levels in the supernatant (Figure 6A). Treatment with ssON, added 4 h after infection with IAV, also had an impact on the viral content detected in the supernatants (Figure 6B). However, the amount of cellular HA-RNA was unchanged in MoDCs treated with ssON 4 h after infection (Supplementary Figure 6B), showing that the ssON did not *per se* interfere with the qRT-PCR and indicating inhibition at the entry level. To get further insight into ssONs capacity to inhibit IAV infection, we investigated whether the stabilization conferred by the chemical PS modification was required, which was found to be the case as the natural PO backbone did not show any antiviral effect (Figure 6C). This finding is in agreement with our previous report showing a short-term transient inhibition of TLR3-mediated responses using the ssON-PO (4). We previously also found that the ssON-mediated TLR3 inhibition required a certain length of the oligonucleotide with a cut-off between 20 and 25 bases (4). Therefore, we subsequently evaluated whether there was an oligonucleotide length requirement for the anti-viral effect. We indeed found that the capacity to limit IAV infection in MoDCs seems to be dependent on the oligonucleotide length (Figure 6C) and reflected the length requirement observed in the inhibition of PolyI:C uptake (Figure 6D). MoDCs were incubated at 37°C with Alexa-488 labeled PolyI:C with or without the different ssONs and the fluorescence signal was quantified by flow cytometry according to methods previously published (4). The fluorescent signal detected at 4°C was used as a control to evaluate the degree of potential surface staining as limited endocytosis occur at 4°C (Figures 6D,F). Accordingly, confocal microscopy confirmed uptake of PolyI:C-Cy3 after incubation at 37°C, which was inhibited in the presence of ssON (Figures 6E–H). Altogether, these data showed that ssON 35 PS has the capacity to reduce IAV infection. Furthermore, that there is a length requirement of the oligonucleotide to confer the anti-IAV inhibitory capacity, which is overlapping with the capacity to inhibit endocytosis of PolyI:C.

### Reduced IAV Load and Disease in Mice Treated With ssON

**Figure 7:**
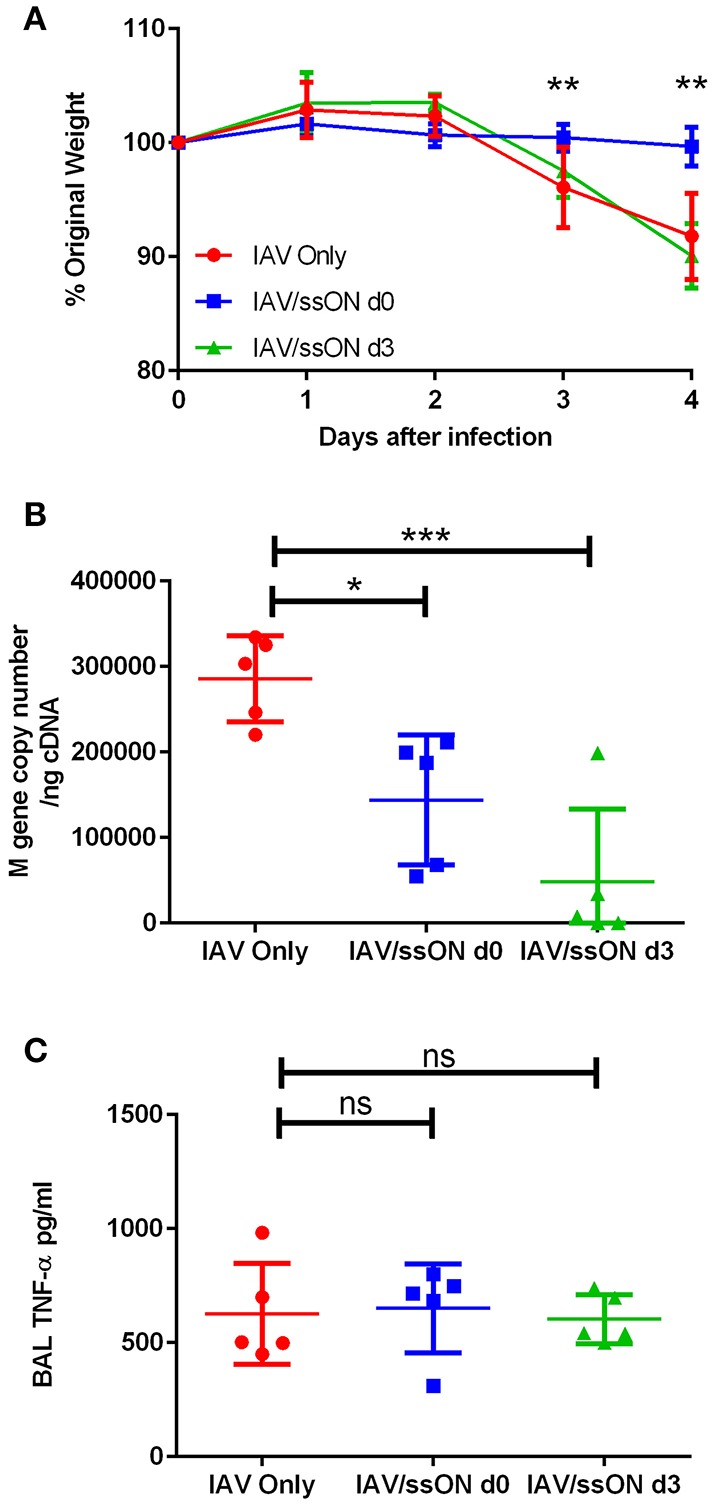
Reduced IAV load in the lungs of mice after treatment with ssON. BALB/c mice were infected intranasally with IAV. One group were untreated (red circles), a second group received 25 μg of ssON (blue squares) intranasally at the same time as the viral challenge and a third group received an intranasal treatment of 25 μg ssON 3 days post-infection (green triangles). **(A)** The mice were weighed daily and subsequently sacrificed day 4 after infection. The **(B)** viral load was measured in the lungs and **(C)** the TNF-α levels present in the bronchoalveolar lavage (BAL) were measured by ELISA. Statistical calculations were made using one-way ANOVA. Data represent one experiment out of two and data for individual mice are shown in **(B,C)**. *P*-value: not significant (ns) *P* > 0.05; *P ≤ 0.05; **P ≤ 0.01; ***P ≤ 0.001.

To investigate the antiviral effect of ssON *in vivo*, mice were infected with IAV by intranasal inoculation, as previously described (22), with or without co-administration of ssON. Alternatively, we administered ssON 3 days post-infection in two independent experiments. The weight of the animals, which is a sensitive parameter of their well-being, started to decline 3 days after IAV exposure (Figure 7A). ssON co-administered with IAV on the day of infection resulted in mice maintaining their starting weight over the first 4 days of infection. Treatment with ssON at day 3 after IAV infection did not affect the weight of the animals (Figure 7A). We found a significantly reduced viral load in the lungs of mice treated with ssON, either co-administered or given intranasally, on day 3 (Figure 7B). However, the viral load bounced back, especially in the group starting treatment on day 3 (Supplementary Figure 7). There was no effect of ssON on the levels of pro-inflammatory TNF-α detected in the airways on day 4 (Figure 7C). Altogether, these data showed an acute reduction of IAV load in mice treated with ssON. Further, co-administration of ssON at the time of infection resulted in a maintained weight of the animals and a clear trend of increased survival monitored up to seven days post-infection (Supplementary Figure 7C). However, starting ssON treatment at day 3 post-infection did not lead to an increased weight in the animals and we did not detect differences in the secretion of TNF-α between the groups of animals. Nevertheless, we detected reduced virus production in the lungs if ssON was administered either before or after initiating virus infection but the effect was not prolonged after a single treatment.

## Discussion

The human population is under the constant threat of the emergence of novel pandemic influenza strains. The last pandemic occurred in 2009, was of swine origin and comprised a new re-assorted H1N1 virus with gene segments originating from different swine and avian influenza viruses (30). There is still a need to develop novel anti-viral prophylactic and/or therapeutic treatments that can combat new emerging viral strains by targeting crucial events in the viral life cycle (3). Influenza A and many other viruses utilize endocytic pathways for entry into cells (17, 18). As we recently showed that ssONs with the length of 25–35 bases inhibit both CME and CDE in MoDC, we here sought to investigate ssON's capacity to limit IAV infection.

Endocytosis has been shown to be a critical step for influenza A virus infection of DCs via DC-SIGN and L-SIGN (17). We detected significant inhibition of IAV infection in MoDC utilizing ssON. We also confirm a length requirement of at least 25 bases to reach a significant effect, which coincided with the length requirement to inhibit uptake of Poly I:C. ssON was not able to completely abolish infection of MoDC *in vitro*, which may be explained by the capacity of influenza viruses to utilize multiple routes for uptake (19, 31). However, by blocking certain endocytic pathways it is conceivable that IAV may be taken up by different routes forcing entry into the cytoplasm and triggering cytoplasmic sensors leading to IFN-production (32). We measured robust induction of IFN-signaling pathways as well as the induction of ISGs including anti-flu genes in the IAV cultures treated with ssON. The transcriptomic analyses conducted 24 h post-infection confirmed induction of ISG signatures concurrent with a previous report suggesting a prolonged innate response in MoDC after infection with pandemic H1N1 as compared with seasonal viruses (33). Additionally, we could not detect extensive induction of genes associated with inflammasomes after IAV infection, which in contrast, was rapidly detected after mucosal SIV infection of macaques using the same gene lists for the MA-plots generated (25). However, future side-by-side studies are required to elaborate on the relative contribution of NLRP3 activation during different viral infections. A limitation of the study is that we did not sort the IAV NP^+^ MoDC prior to RNA isolation and such study may reveal more distinct signaling pathways related to the IAV infection.

The RNA sequencing data revealed that IAV-infected cultures treated with PolyI:C resulted in additional top-up of many genes, suggesting that the MoDC were susceptible to external TLR3 activation. However, ssON treatment did not impact the baseline gene expression induced by the IAV infection, indicating that the cultures were intrinsically avoid of TLR3 ligands. To get an overall picture of global gene expression changes, we conducted pathway analyses and compared cultures kept in medium alone with those infected with IAV and treated with ssON, with the goal to identify possible signaling pathways affected by the ssON treatment. Cultures treated with ssON, without any further stimuli, did not show any significant changes in the transcriptome or proteome (4). IAV infection induced a number of pathways including “Interferon signaling” and innate immune responses associated with the “Crosstalk between Dendritic cells and Natural Killer Cells.” “Caveolar-mediated Endocytosis Signaling” was among the top 8 pathways after IAV infection. The IPA pathways identified in the IAV/ssON treated cultures included several innate immune signaling pathways such as “Interferon Signaling,” “Activation of IRF by Cytosolic Pattern Recognition Receptors,” “Role of Pattern Recognition Receptors in Recognition of Bacteria and Viruses” and “Role of RIG1-like Receptors in Antiviral Innate Immunity.” One intriguing pathway was associated with cholesterol biosynthesis, which showed a significant down-regulation of the “Superpathway of Cholesterol Biosynthesis.” A recent paper demonstrated that mice transduced with an adenoviral vector expressing a long non-coding RNA (termed *LeXis*) showed reduced serum cholesterol in both the LDL and HDL fractions (28). Pathway analysis of global gene expression revealed that the cholesterol biosynthetic pathway was strongly down-regulated in *LeXis*-transduced livers (28). It remains to be elucidated whether the ssON described here also has an impact on cholesterol levels. It was previously shown that TLR3 activation blocks the induction of the liver X receptors (LXR), which are regulators of cholesterol metabolism (34). The cross-talk between LXR and TLR3 signaling was shown to be mediated by IRF3, highlighting a common mechanism whereby viral pathogens may modulate cholesterol metabolism (34).

TLR3 activation leads to phosphorylation of both IRF3 and NFκB. We previously showed that a CpG containing ssON prevented activation of both NFκB and IRF3 in MoDC, revealing a block in the signaling cascade (35). By inhibiting of uptake of ligands destined for TLR3/4/7-signaling endosomes, ssON inhibits downstream induction of pro-inflammatory cytokines in MoDC, epithelial cells and keratinocytes (4). As TLR3/4/7 responses may also contribute to influenza pathogenesis by causing excessive pro-inflammatory cascades (12), we also investigated the capacity of ssON to limit cytokine production upon TLR3 activation of IAV-infected MoDC. We could indeed confirm inhibition of TLR3-mediated cytokine responses (IL-6, IL-12p40, IL-12p70, IFN-α, and IL-29) by ssON. Hence, ssON was able to inhibit the top-up of cytokine release caused by PolyI:C, but maintained a baseline cytokine secretion which was likely induced by the IAV infection *per se*. The relatively low levels of cytokines and IFNs that we detected after H1N1 infection in MoDC *in vitro* are in agreement with a previous report (36). However, some patients infected with the pandemic H1N1 2009 virus displayed enhanced production of IL-6, IL-8, and CCL2 (37), most likely reflecting the more complex multicellular environment *in vivo*. Altogether, our findings show that ssON can inhibit cytokine/chemokines induced by dsRNA contributing to excessive inflammation but spare IAV induced ISG responses in MoDC.

We performed intracellular staining using an anti-NP mAb to be able to study both infected and non-infected cells in the cultures. Depending on the influenza A subtype, the virus may (15) or may not (16) trigger innate responses leading to expression of co-stimulatory molecules. We here found that pandemic H1N1 A/Cal/07/2009 inhibited up-regulation of CD80 and CD86 expression, specifically in the NP^+^ MoDC, and that the culture condition promoted their up-regulation in neighboring cells. We furthermore found that IAV^+^ MoDC were refractory to PolyI:C mediated induction of maturation. These data are consistent with previous findings that the NS1 protein of influenza virus can prevent induction of co-stimulatory molecules in MoDC (38), which is likely to impair their antigen presenting capacity. We found a significant but modest induction of co-stimulatory molecules in the neighboring non-infected MoDC, which could be boosted further by PolyI:C treatment. Notably, the top up maturation by PolyI:C in IAV^−^ MoDC was inhibited by ssON, in agreement with our previous finding showing ssON's capacity to inhibit endosomal TLR3 signaling in MoDC. However, ssON did not block the maturation caused by the IAV culture condition *per se*, suggesting that it was not dependent on TLR signaling from endosomes. MoDC can be induced to mature after exposure to different cytokines and IFNs (39). Both IFN-α and IL-29 were detected in the IAV cultures, and it is conceivable that the inflammatory milieu in the culture contributed to the bystander MoDC maturation.

There are limitations of using MoDC *in vitro*, however, a previous study conducting transcriptomic analyses of MoDC infections *in vitro* with several influenza strains reported a good correspondence with gene signatures obtained from PBMC of infected individuals (33). The reason why we did not extend the *in vitro* analyses to respiratory epithelial cells is due to the notion that influenza infection of these cells requires addition of trypsin to the cultures (40). The sialic acid (SA) has been identified as the primary attachment site on the cell surface that interacts with the viral HA binding site (41, 42). Subsequent to the interaction with HA and SA-containing receptors, the virus uses CME or CDE for cellular entry (19). *In vivo*, there are trypsins available at the epithelial surface (41, 42), while it has to be added exogenously for *in vitro* infections of epithelial cells. We found in initial experiments that trypsin treatment abolished ssONs capacity to interfere with endocytosis (data not shown). Thus, we performed the *in vitro* experiments with MoDC, which are susceptible to influenza A without the addition of trypsin.

We here also report on ssONs capacity to inhibit IAV infection in mice upon intranasal challenge. We used an established H1N1 A/England/195/2009 murine infection model, which is derived from a clinical isolate, rather than the H1N1 A/Cal/07/2009 IAV that was used for the *in vitro* experiments (22), this was because it was an established model and comparable to other studies, the two viruses are antigenically the same and differ only marginally. The results show that the antiviral activity is not strictly dependent on the IAV sequence, which was expected as the ssON is not complementary to any known IAV sequence. It remains to be evaluated whether ssON has a broad anti-IAV activity and if other viruses can be inhibited as well. We detected both reduced viral load in the lungs and maintained weight in animals that received ssON concomitant with IAV. However, when ssON was administered 3 days after the viral challenge, we detected significantly reduced viral load in the lungs but no change in the overall progressive pathogenesis as measured by weight loss. Even though we found reduced pro-inflammatory cytokine production *in vitro* by adding ssON in IAV-infected MoDC, we did not measure any reduced TNF-α production in the lungs of ssON treated mice. We previously showed that ssON inhibits endosomal TLR3/4/7 activation but leaves cells capable of responding to other ligands/pathways. It is therefore conceivable that the complex cellular environment created *in vivo* upon IAV infection in mice engaged alternative pathways leading to pro-inflammatory cytokines. Previous studies have shown the detrimental effects of MyD88 dependent TLR4 signaling in the pathogenesis of IAV in mice via the DAMP molecule S100A9 (43). As we previously indicated that ssON does not inhibit the MyD88-dependent pathway but only the TRIF-dependent pathway upon LPS stimulation, it is not likely that ssON could have any effect *in vivo* on MyD88-dependent signaling.

Altogether, we here show that treatment with a 35 bases long PS-ssON reduced IAV infection of human primary MoDC *in vitro* and led to inhibition of TLR3-mediated cytokine responses. The capacity of ssON to limit IAV infection required a length of at least 20–25 bases, which coincided with the capacity to inhibit endosomal uptake of dsRNA (PolyI:C). We further provide evidence of reduced viral replication *in vivo* in mice treated with ssON, supporting further studies investigating ssONs capacity to limit viral infections.

## Data Availability

RNA-seq data have been deposited in the ArrayExpress database at EMBL-EBI (www.ebi.ac.uk/arrayexpress) under accession number E-MTAB-7803.

## Ethics Statement

The animal study was reviewed and approved by the Animal Welfare and Ethical Review Board (AWERB) at Imperial College London.

## Author Contributions

CP, AD, JB, SP, CA and DB performed the experimental studies and analyzed data. PJ, A-LS, and JA provided the technical input and data analysis. VC and RL provided and grew the influenza strains. JL, JT, and A-LS designed the studies. CP, AD, JB, and A-LS wrote the paper.

### Conflict of Interest Statement

PJ, AD, and A-LS are authors of patent applications related to the work and are shareholders of TIRmed Pharma. The remaining authors declare that the research was conducted in the absence of any commercial or financial relationships that could be construed as a potential conflict of interest.

## Abbreviations

BAL: bronchoalveolar lavage
CME: clathrin-mediated endocytosis
CDE: caveolin-dependent endocytosis
DAMPs: damage-associated molecular patterns
DC: dendritic cell
HI: heat-inactivated
IAV: influenza A H1N1
IFN: interferon
ISGs: interferon stimulated genes
h: hour
HA: hemagglutinin
MoDC: human monocyte-derived dendritic cells
MOI: multiplicity of infection
NP: IAV nucleoprotein
IPA: ingenuity pathway analysis
LXR: liver X receptors
pDCs: plasmacytoid dendritic cell
p.i.: post-infection
PRR: pattern recognition receptors
PO: phosphodiester
PS: phosphorothioate
qRT-PCR: quantitative RT-PCR
mAb: monoclonal antibody
SA: sialic acid
ssON: single-stranded oligonucleotides
TLR: toll-like receptor.

## References

[1] Ruuskanen O Lahti E Jennings LC Murdoch DR. Viral pneumonia. Lancet. (2011) 377:1264–75. 10.1016/S0140-6736(10)61459-6 21435708 PMC7138033

[2] Tscherne DM Garcia-Sastre A. Virulence determinants of pandemic influenza viruses. J Clin Invest. (2011) 121:6–13. 10.1172/JCI44947 21206092 PMC3007163

[3] van de Wakker SI Fischer MJE Oosting RS. New drug-strategies to tackle viral-host interactions for the treatment of influenza virus infections. Eur J Pharmacol. (2017) 809:178–90. 10.1016/j.ejphar.2017.05.038 28533172

[4] Jarver P Dondalska A Poux C Sandberg A Bergenstrahle J Skold AE. Single-stranded nucleic acids regulate TLR3/4/7 activation through interference with clathrin-mediated endocytosis. Sci Rep. (2018) 8:15841. 10.1038/s41598-018-33960-4 30367171 PMC6203749

[5] Short KR Kasper J van der Aa S Andeweg AC Zaaraoui-Boutahar F Goeijenbier M. Influenza virus damages the alveolar barrier by disrupting epithelial cell tight junctions. Eur Respir J. (2016) 47:954–66. 10.1183/13993003.01282-2015 26743480

[6] Iwasaki A Pillai PS. Innate immunity to influenza virus infection. Nat Rev Immunol. (2014) 14:315–28. 10.1038/nri3665 24762827 PMC4104278

[7] Schneider WM Chevillotte MD Rice CM. Interferon-stimulated genes: a complex web of host defenses. Annu Rev Immunol. (2014) 32:513–45. 10.1146/annurev-immunol-032713-120231 24555472 PMC4313732

[8] Pandey S Kawai T Akira S. Microbial sensing by Toll-like receptors and intracellular nucleic acid sensors. Cold Spring Harb Perspect Biol. (2014) 7:a016246. 10.1101/cshperspect.a016246 25301932 PMC4292165

[9] Kurts C Robinson BW Knolle PA. Cross-priming in health and disease. Nat Rev Immunol. (2010) 10:403–14. 10.1038/nri2780 20498667

[10] Vangeti S Yu M Smed-Sorensen A. Respiratory mononuclear phagocytes in human influenza A virus infection: their role in immune protection and as targets of the virus. Front Immunol. (2018) 9:1521. 10.3389/fimmu.2018.01521 30018617 PMC6037688

[11] Jensen S Thomsen AR. Sensing of RNA viruses: a review of innate immune receptors involved in recognizing RNA virus invasion. J Virol. (2012) 86:2900–10. 10.1128/JVI.05738-11 22258243 PMC3302314

[12] Pulendran B Maddur MS. Innate immune sensing and response to influenza. Curr Top Microbiol Immunol. (2015) 386:23–71. 10.1007/82_2014_405 25078919 PMC4346783

[13] Pichlmair A Schulz O Tan CP Naslund TI Liljestrom P Weber F. RIG-I-mediated antiviral responses to single-stranded RNA bearing 5'-phosphates. Science. (2006) 314:997–1001. 10.1126/science.1132998 17038589

[14] Le Goffic R Balloy V Lagranderie M Alexopoulou L Escriou N Flavell R. Detrimental contribution of the Toll-like receptor (TLR)3 to influenza A virus-induced acute pneumonia. PLoS Pathog. (2006) 2:e53. 10.1371/journal.ppat.0020053 16789835 PMC1475659

[15] Chin R Earnest-Silviera L Gordon CL Olsen K Barr I Brown LE. Impaired dendritic cell maturation in response to pandemic H1N109 influenza virus. J Clin Virol. (2013) 56:226–31. 10.1016/j.jcv.2012.11.009 23218952

[16] Smed-Sorensen A Chalouni C Chatterjee B Cohn L Blattmann P Nakamura N. Influenza A virus infection of human primary dendritic cells impairs their ability to cross-present antigen to CD8 T cells. PLoS Pathog. (2012) 8:e1002572. 10.1371/journal.ppat.1002572 22412374 PMC3297599

[17] Gillespie L Roosendahl P Ng WC Brooks AG Reading PC Londrigan SL. Endocytic function is critical for influenza A virus infection via DC-SIGN and L-SIGN. Sci Rep. (2016) 6:19428. 10.1038/srep19428 26763587 PMC4725901

[18] Cossart P Helenius A. Endocytosis of viruses and bacteria. Cold Spring Harb Perspect Biol. (2014) 6:a016972. 10.1101/cshperspect.a016972 25085912 PMC4107984

[19] Lakadamyali M Rust MJ Zhuang X. Endocytosis of influenza viruses. Microbes Infect. (2004) 6:929–36. 10.1016/j.micinf.2004.05.002 15310470 PMC2715838

[20] Svensson MJ Lind I Wirgart BZ Ostlund MR Albert J Performance of the simplexa flu A/B & RSV direct kit on respiratory samples collected in saline solution. Scand J Infect Dis. (2014) 46:825–31. 10.3109/00365548.2014.946444 25195649

[21] Elderfield RA Watson SJ Godlee A Adamson WE Thompson CI Dunning J. Accumulation of human-adapting mutations during circulation of A(H1N1)pdm09 influenza virus in humans in the United Kingdom. J Virol. (2014) 88:13269–83. 10.1128/JVI.01636-14 25210166 PMC4249111

[22] Groves HT McDonald JU Langat P Kinnear E Kellam P McCauley J. Mouse models of influenza infection with circulating strains to test seasonal vaccine efficacy. Front Immunol. (2018) 9:126. 10.3389/fimmu.2018.00126 29445377 PMC5797846

[23] Siggins MK Gill SK Langford PR Li Y Ladhani SN Tregoning JS. PHiD-CV induces anti-Protein D antibodies but does not augment pulmonary clearance of non-typeable *Haemophilus influenzae* in mice. Vaccine. (2015) 33:4954–61. 10.1016/j.vaccine.2015.07.034 26212006

[24] Hartmann BM Albrecht RA Zaslavsky E Nudelman G Pincas H Marjanovic N. Pandemic H1N1 influenza A viruses suppress immunogenic RIPK3-driven dendritic cell death. Nat Commun. (2017) 8:1931. 10.1038/s41467-017-02035-9 29203926 PMC5715119

[25] Barouch DH Ghneim K Bosche WJ Li Y Berkemeier B Hull M. Rapid inflammasome activation following mucosal SIV infection of rhesus monkeys. Cell. (2016) 165:656–67. 10.1016/j.cell.2016.03.021 27085913 PMC4842119

[26] Park HS Liu G Thulasi Raman SN Landreth SL Liu Q Zhou Y. NS1 protein of 2009 pandemic influenza A virus inhibits porcine NLRP3 inflammasome-mediated interleukin-1 beta production by suppressing ASC ubiquitination. J Virol. (2018) 92:e00022–18. 10.1128/JVI.00022-18 29386291 PMC5874408

[27] Tate MD Mansell A An update on the NLRP3 inflammasome and influenza: the road to redemption or perdition? Curr Opin Immunol. (2018) 54:80–5. 10.1016/j.coi.2018.06.005 29986838

[28] Sallam T Jones MC Gilliland T Zhang L Wu X Eskin A. Feedback modulation of cholesterol metabolism by the lipid-responsive non-coding RNA LeXis. Nature. (2016) 534:124–8. 10.1038/nature17674 27251289 PMC4896091

[29] Lazear HM Nice TJ Diamond MS. Interferon-lambda: immune functions at barrier surfaces and beyond. Immunity. (2015) 43:15–28. 10.1016/j.immuni.2015.07.001 26200010 PMC4527169

[30] York I Donis RO. The 2009 pandemic influenza virus: where did it come from, where is it now, and where is it going? Curr Top Microbiol Immunol. (2013) 370:241–57. 10.1007/82_2012_221 22638836

[31] Rossman JS Leser GP Lamb RA. Filamentous influenza virus enters cells via macropinocytosis. J Virol. (2012) 86:10950–60. 10.1128/JVI.05992-11 22875971 PMC3457176

[32] Chow KT Gale M Jr Loo YM. RIG-I and other RNA sensors in antiviral immunity. Annu Rev Immunol. (2018) 36:667–94. 10.1146/annurev-immunol-042617-053309 29677479

[33] Hartmann BM Thakar J Albrecht RA Avey S Zaslavsky E Marjanovic N. Human dendritic cell response signatures distinguish 1918, pandemic, and seasonal H1N1 influenza viruses. J Virol. (2015) 89:10190–205. 10.1128/JVI.01523-15 26223639 PMC4580178

[34] Castrillo A Joseph SB Vaidya SA Haberland M Fogelman AM Cheng G. Crosstalk between LXR and toll-like receptor signaling mediates bacterial and viral antagonism of cholesterol metabolism. Mol Cell. (2003) 12:805–16. 10.1016/S1097-2765(03)00384-8 14580333

[35] Skold AE Hasan M Vargas L Saidi H Bosquet N Le Grand R. Single-stranded DNA oligonucleotides inhibit TLR3-mediated responses in human monocyte-derived dendritic cells and *in vivo* in cynomolgus macaques. Blood. (2012) 120:768–77. 10.1182/blood-2011-12-397778 22700721

[36] Osterlund P Pirhonen J Ikonen N Ronkko E Strengell M Makela SM. Pandemic H1N1 2009 influenza A virus induces weak cytokine responses in human macrophages and dendritic cells and is highly sensitive to the antiviral actions of interferons. J Virol. (2010) 84:1414–22. 10.1128/JVI.01619-09 19939920 PMC2812319

[37] Lee N Wong CK Chan PK Chan MC Wong RY Lun SW. Cytokine response patterns in severe pandemic 2009 H1N1 and seasonal influenza among hospitalized adults. PLoS ONE. (2011) 6:e26050. 10.1371/journal.pone.0026050 22022504 PMC3192778

[38] Fernandez-Sesma A Marukian S Ebersole BJ Kaminski D Park MS Yuen T. Influenza virus evades innate and adaptive immunity via the NS1 protein. J Virol. (2006) 80:6295–304. 10.1128/JVI.02381-05 16775317 PMC1488970

[39] Castiello L Sabatino M Jin P Clayberger C Marincola FM Krensky AM. Monocyte-derived DC maturation strategies and related pathways: a transcriptional view. Cancer Immunol Immunother. (2011) 60:457–66. 10.1007/s00262-010-0954-6 21258790 PMC3086891

[40] Eisfeld AJ Neumann G Kawaoka Y. Influenza A virus isolation, culture and identification. Nat Protoc. (2014) 9:2663–81. 10.1038/nprot.2014.180 25321410 PMC5619698

[41] Laporte M Naesens L. Airway proteases: an emerging drug target for influenza and other respiratory virus infections. Curr Opin Virol. (2017) 24:16–24. 10.1016/j.coviro.2017.03.018 28414992 PMC7102789

[42] Garten W Braden C Arendt A Peitsch C Baron J Lu Y. Influenza virus activating host proteases: identification, localization and inhibitors as potential therapeutics. Eur J Cell Biol. (2015) 94:375–83. 10.1016/j.ejcb.2015.05.013 26095298

[43] Tsai SY Segovia JA Chang TH Morris IR Berton MT Tessier PA. DAMP molecule S100A9 acts as a molecular pattern to enhance inflammation during influenza A virus infection: role of DDX21-TRIF-TLR4-MyD88 pathway. PLoS Pathog. (2014) 10:e1003848. 10.1371/journal.ppat.1003848 24391503 PMC3879357

