# Supplementary material for: A Single-Stranded Oligonucleotide Inhibits Toll-Like Receptor 3 Activation and Reduces Influenza A (H1N1) Infection
Source: Front Immunol. 2019 Sep 12;10:2161. doi: 10.3389/fimmu.2019.02161 (PMC6751283; doi:10.3389/fimmu.2019.02161)
Supplement: Supplementary file 1 [file Data_Sheet_1.pdf]

Supplementary information for Candice Poux et al "A Single-Stranded Oligonucleotide Inhibits Toll-Like Receptor 3 Activation and Reduces Influenza A (H1N1) Infection" containing 7 figures and 4 Tables.

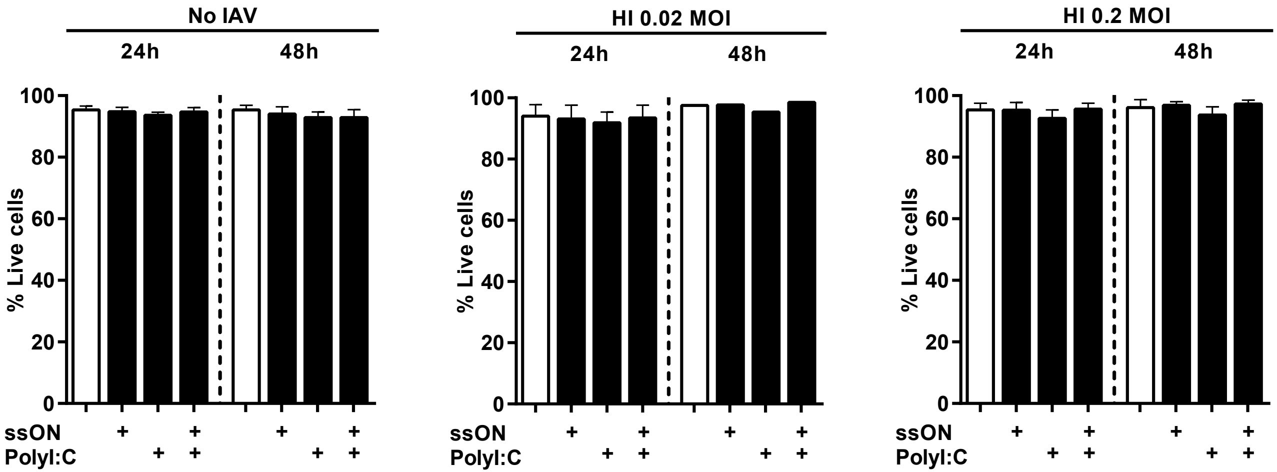

Supplementary Figure 1. Viability data after exposure to HI IAV.
No increased cell death was observed in MoDC treated with PolyI:C or ssON and cultured for 24h or 48h without IAV infection or in the presence of HI IAV using 0.02 or 0.2MOI. Mean ±SEM. For No IAV (n=10 at 24h), at least three independent experiments were performed and the HI experiments were performed in two-four independent experiments.

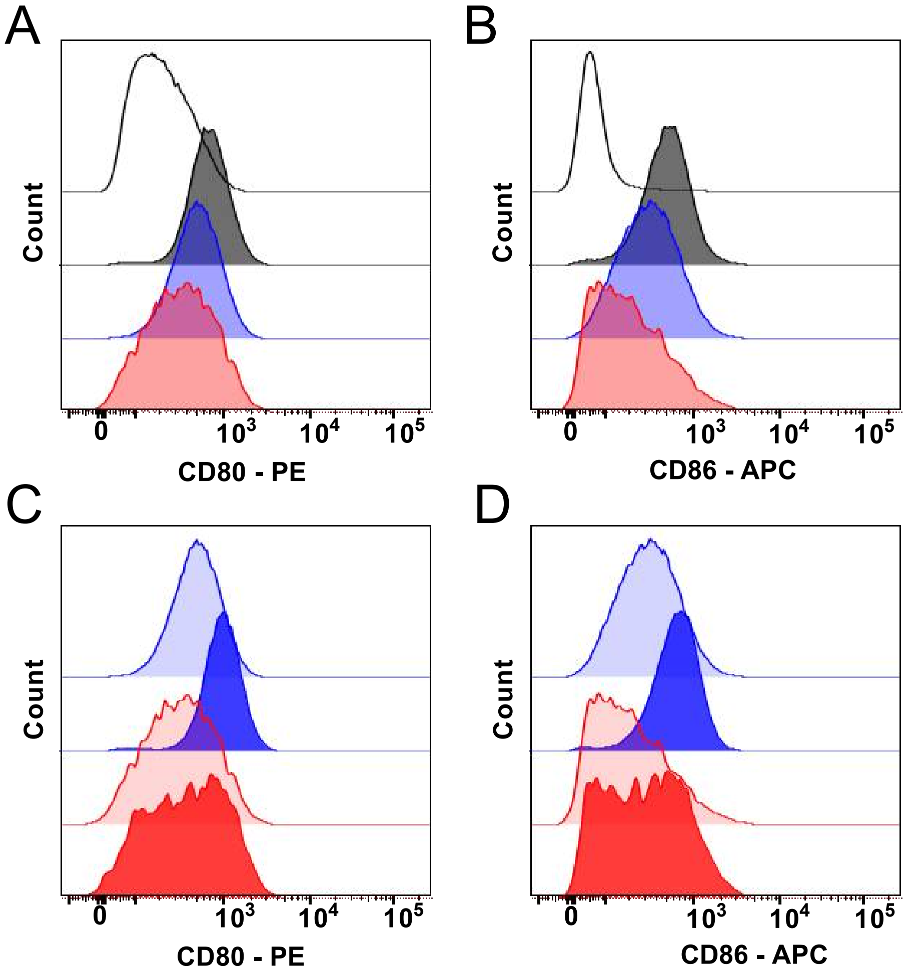

Supplementary Figure 2. Example analysis of CD80 and CD86 expression in MoDC. Representative flow cytometry staining for the analysis of co-stimulatory molecules in MoDC is shown using overlaying histograms for CD80 (A and C) and CD86 (B and D) expression in gated live MoDC from one donor 24 hours post treatment with or without IAV (MOI of 0.2). For (A) and (B), medium treated cells are represented as a black unfilled histogram, PolyI:C treated cells as a black filled histogram, IAV treated NP- cells as a blue histogram, and IAV treated NP+ cells as a red histogram. For (C) and (D), MoDC were infected with IAV and then treated with or without PolyI:C (25 µg/ml); NP- cells are represented in blue, NP+ cells are represented in red, and cells further treated with PolyI:C are depicted using solid histograms.

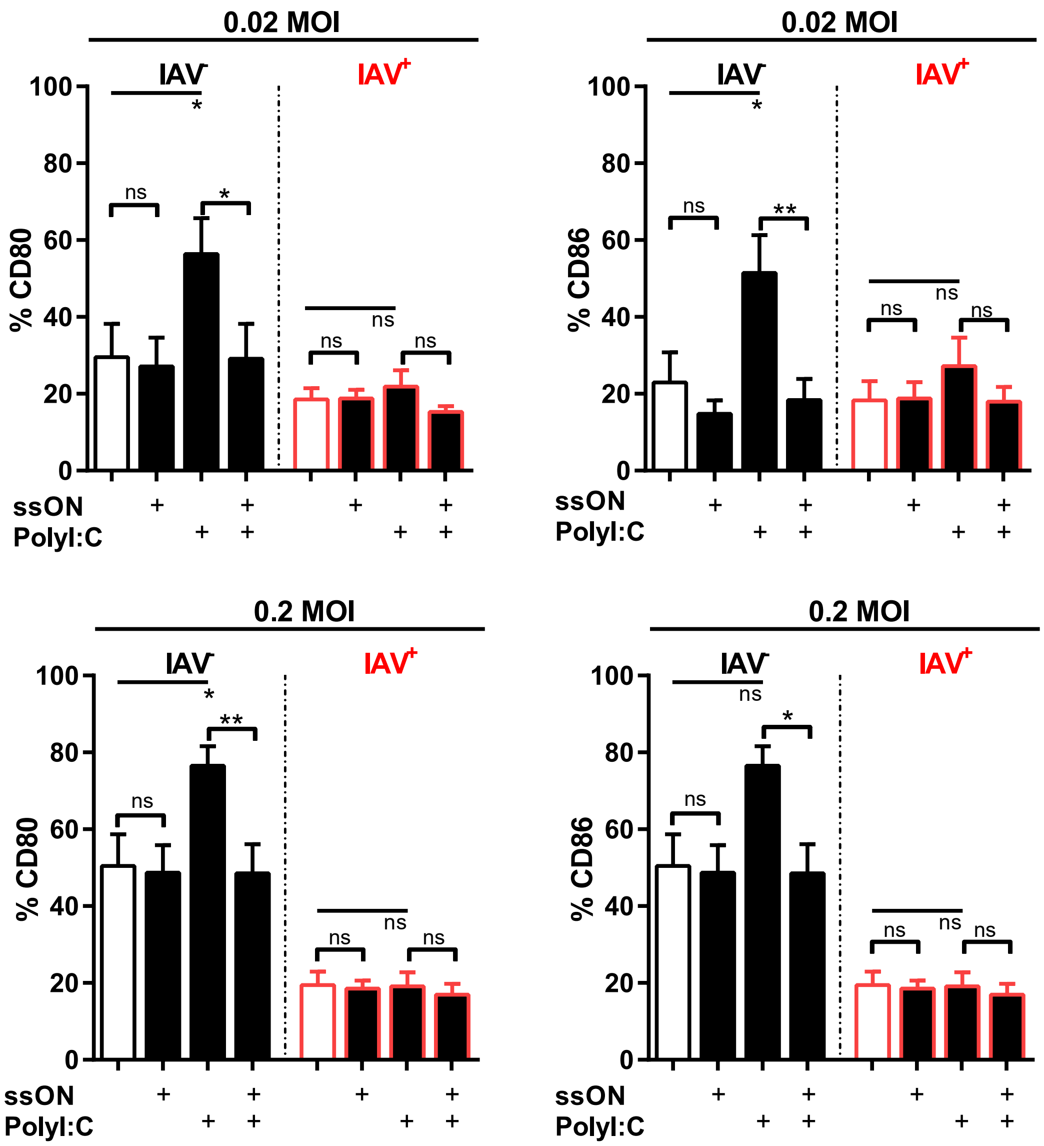

Supplemenatry Figure 3. Expression of CD80 and CD86 in IAV NP^{+} and IAV NP^{-} MoDC.
MoDC were mock or IAV-infected at indicated MOI for 4h followed by addition of ligands. Statistical analysis were performed in comparison with mock infection (Medium) using one-way ANOVA with Dunn´s multiple comparison test (alpha 0.05) and indicated with a line. Calculations made between two groups using two-tailed Mann-Whitney test were depicted with a bar. *P*-value: not significant (ns) $P > 0.05$; * $P < 0.05$; ** $P < 0.01$; *** $P < 0.001$. Frequencies of CD80 and CD86 expressing cells were measured by flow cytometry after gating on live non-infected and live IAV-infected NP+ (red line) MoDC (gating shown in Figure 1E and Supplementary Figure 2). Mean ±SEM with n=8 (24h) .

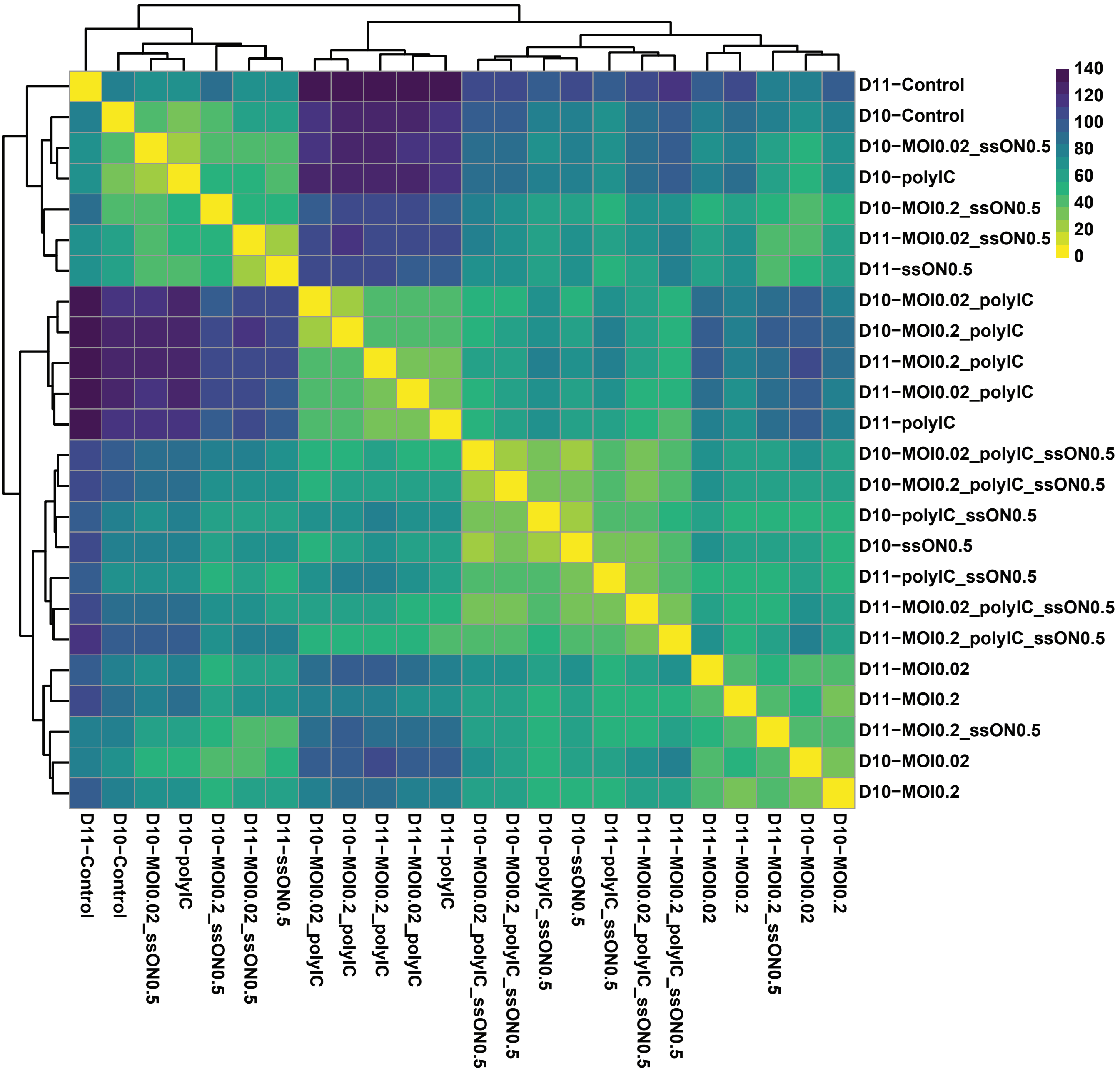

Supplementary Figure 4. Heatmap of sample-to-sample distances.
The raw count data was transformed according to the regularized logarithm (rlog) method provided in the DESeq2 R package. A sample-to-sample distance matrix was calculated on the transformed counts with the dist function within R and the hierarchical clustering was based on the output. D10/D11 = Donor10/Donor11.

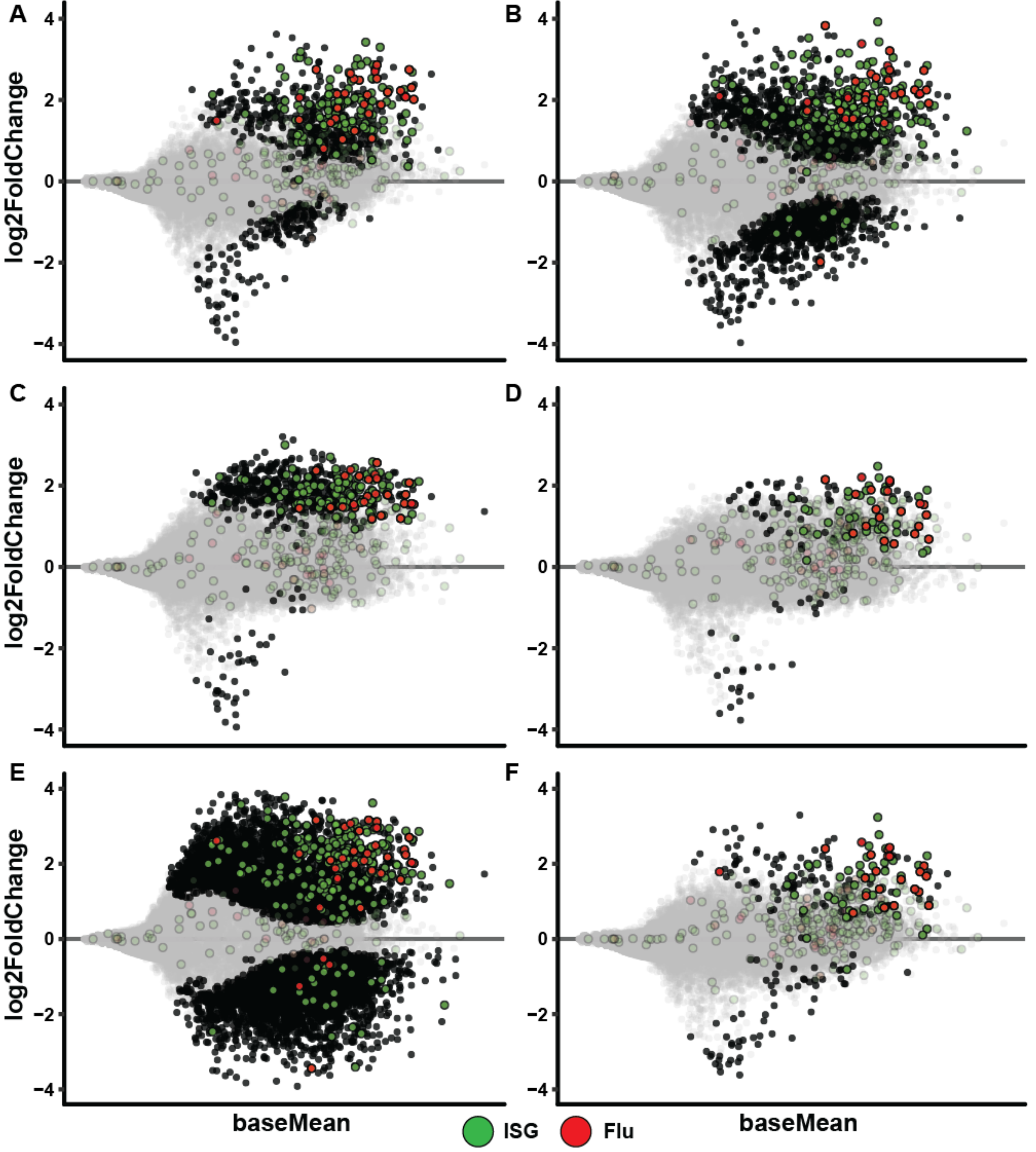

Supplementary Figure 5. Virus infected MoDC display up-regulation of ISGs.
Each comparison includes four samples. Genes with adjusted p-value below 0.05 are fully coloured (ISG = green, Flu = red, rest = black). (A) Control vs IAV 0.02MOI (B) Control vs IAV 0.2MOI (C) Control vs PolyI:C (D) Control vs ssON (E) Control vs IAV 0.2MOI + PolyI:C stimulation (F) Control vs IAV 0.2MOI + ssON stimulation

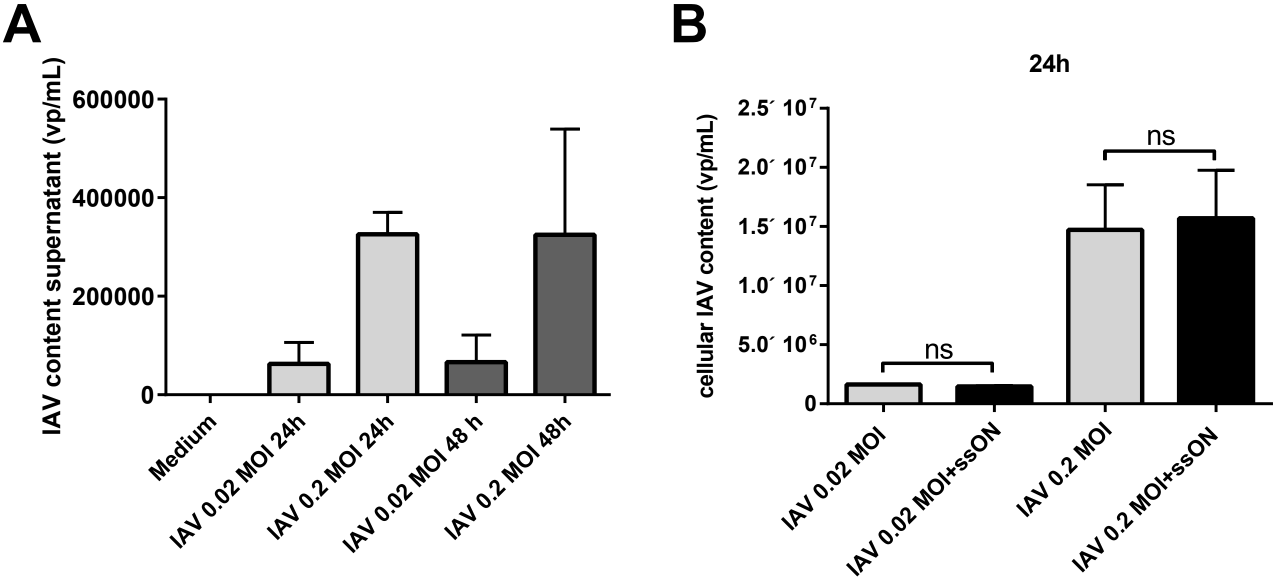

Supplementary Figure 6. Dose-response and kinetics of IAV infection in MoDC.
MoDC were mock or IAV-infected at indicated MOI for 4h or kept in medium. Viral content in the supernatant was determined by qRT-PCR for the HA gene after 24h and 48 h. Four individual donors were evaluated in triplicates. Mean+SEM (A). MoDC were IAV-infected at indicated MOI for 4h, washed and then incubated without or with ssON for 24h before harvesting the cells and measuring cellular viral content by qRT-PCR for the HA gene (B). Non-parametric statistical analyses were used. Mean±SEM with n=6 analysed in triplicates for IAV 0.2MOI .

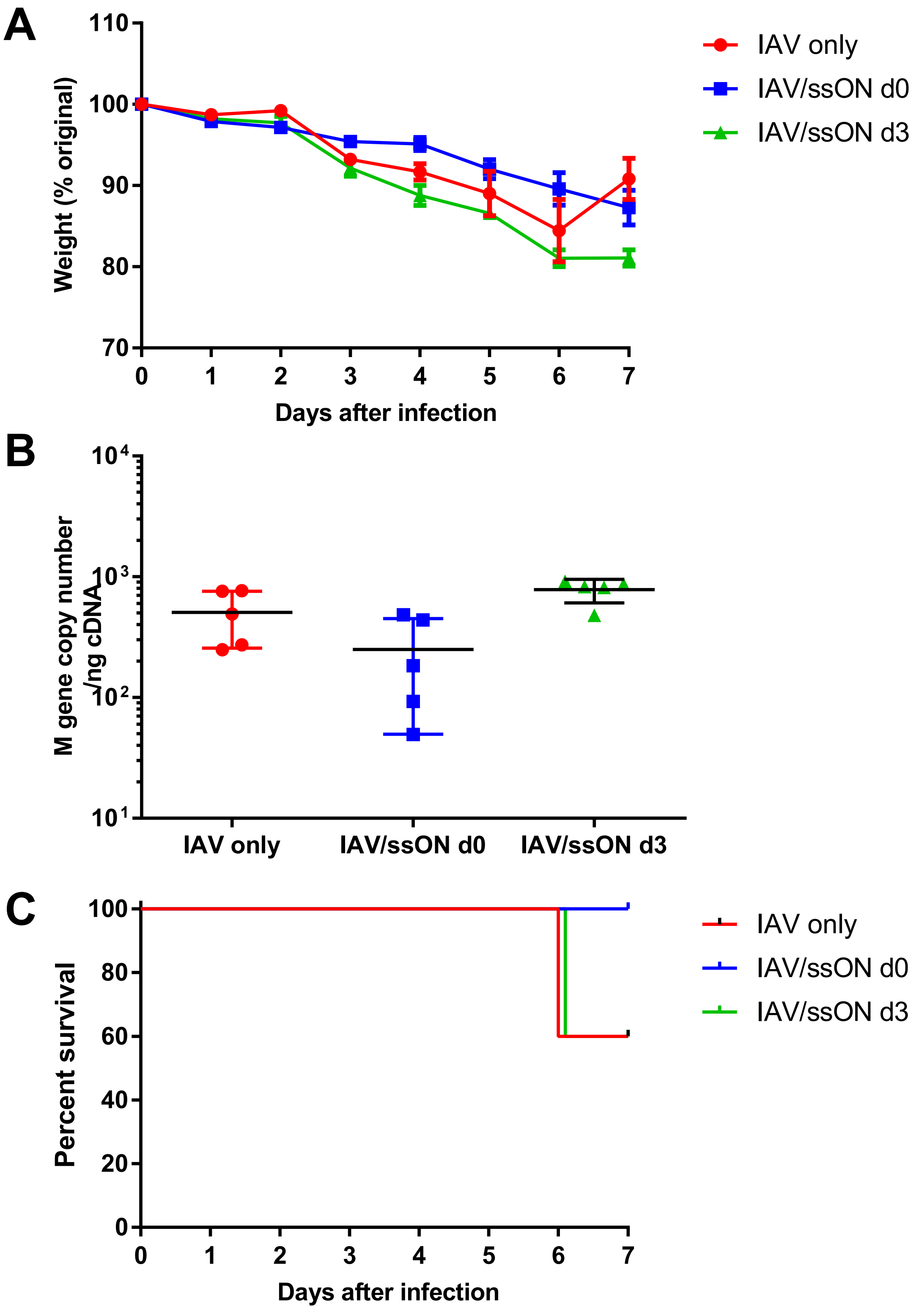

Supplemetary Figure 7. Trend of increased survival in mice treated with ssON the same day as IAV infection. BALB/c mice were infected intranasally with IAV. One group were untreated (red circles), a second group received 25 μg of ssON (blue squares) intranasally at the same time as the viral challenge and a third group received an intranasal treatment of 25 μg ssON three days post-infection (green triangles). (A) The mice were weighed daily and the surviving animals were subsequently sacrificed day 7 after infection. The (B) viral load was measured in the lungs and (C) shows the survival of the animals up to seven days post-infection.

Supplementary Table 1. List of ISG genes

MAP3K14
mean-HA
ELF1
IFIH1
IFITM3
PMM2
DDX58
MX1
FAM46C
TBX3
IRF2
SCO2
BST2
SERPINE1
HERC6
IRF7
MOV10
CCL5
UPP2
B2M
MARCK
CRY1
LAMP3
IL28RA
RIPK2
MT1L
XAF1
MAFF
CD274
TAP2
IFITM2
TNFRSF10A
SAT1
IRF1
SLC25A28
IFI44
TRIM34
NPAS2
GBP5
TNFSF10
GPX2
THBD
EIF3EIP
MX2
CSDA
IRF9
SLC15A3
PTMA
KIAA0040

ARHGEF3
MYD88
C2orf31
XRN
CCL2
CTCFL
IFI35
FLJ23556
DUSP5
ENPP1
HERC5
LMO2
RBCK1
CCL4
SP110
TRIM56
HLA-C
TDRD7
MAFF
LGALS9
LAP3
THOC4
TRIM21
PFKFB3
GMPR
CX3CL1
FER1L3
DEFB1
SAMD4A
CXCL11
ADM
SPTLC2
FLT1
ULK4
ABLIM3
LGMN
HES4
GBP4
RNF19B
IFIT5
FNDC4
CRP
ZNF313
MT1M
SMAD3
PRAME
AHNAK2
LEPR
MT1H
NCOA3

C9ofr19
ABCA9
MTHFD2L
RBM25
SIRPA
HSH2D
APOL1
NCF1
LGALS3
RIPK2
CFB
ERLIN1
CCL19
TNK2
BCL3
MT1G
PXK
ABTB2
ODC1
MAP3K5
STAP1
EHD4
CXCL10
SP100
TLR7
TRIM5
ISG20
GK
ARG2
PFKFB3
CCDC75
CREB3L3
WHDC1
SECTM1
IFI16
C6orf150
CMAH
PLSCR1
MASTL
C4orf32
SOCS2
SAA1
RBM43
ANKFY1
MCOLN
HPSE
AQP9
SLC16A1
FLJ11286
USP18

FAM134B
ATF3
ZNF385B
MCL1
BCL2L14
CLEC4D
SLC25A30
PPM1K
MICB
NRN1
BLZF1
DDX60
HCP5
HESX1
BIRC3
PARP12
CCL8
NOS2A
FBXO6
CD74
SERPINB9
NDC80
RSAD2
AGPAT9
ZNF295
STAT3
TRIM25
ARNTL
GZMB
N4BP1
OGFR
C9orf91
LAMP3
GCH1
STAT1
CES1
RASSF4
CDKN1A
S100A8
EPSTI1
PCTK2
CD80
PI4K2B
P2RY6
DDIT4
IL15RA
RAB27A
CD163
TRAFD1
FUT4

EIF2AK2
GJA4
TAGAP
CHMP5
JUNB
PSMB9
PIM3
FAM70A
LOC400759
DTX3L
FKBP5
PABPC4
PBEF1
LY6E
VAMP5
TGFB1
MS4A4A
GBP2
CPT1A
CD9
RGL-1
SCARB2
PDGFRL
UBE2L6
OPTN
EPAS1
APOL2
PADI2
AKT3
ZC3HAV1
LRG1
PCTK3
NAPA
ANGPTL1
LIPA
CASP7
DHX58
STARD5
MAB21L2
BATF2
SSBP3
TMEM51
TRIM38
DNAPTP6
APOL6
ADAMDEC1
GEM
IFIT3
TNFAIP3
DCP1A

SAMHD1
IFI44L
CCR1
KIAA1618
SOCS1
CD69
CXCL9
SIGLEC1
INDO
ISG15
AIM2
UNC84B
ETV6
OAS3
PSMB8
C5orf27
C22orf28
IFI27
NUP50
CNP
FLJ39739
HLA-F
TNFAIP6
IL17RB
DYNLT1
HEG1
NOD2
FAM125B
PDK1
TCF7L2
HK2
RARRES3
NT5C3
UNC93B1
PNRC1
PUS1
TIMP1
IL1R
LINCR
SQLE
RASGEF1B
IFNGR1
VEGFC
GAK
DDX60
MAFF
IFI30
ARHGAP17
HLA-G
TLR3

EXT1
PHF15
BTN3A3
GALNT2
FCGR1A
IL15
FNDC3B
CLEC4A
TMEM49
MT1F
CLEC2B
SLFN5
NFIL3
CEACAM1
CCDC92
CCNA1
GBP3
IL6ST
ATP10D
ADFP
GLRX
OASL
GTPBP2
IRF2
RGS1
STAT2
CYP1B1
DDX3X
C10orf10
HSPA6
PNPT1
RNF24
OAS1
RPL22
SLC1A1
AXUD1
CCDC109B
NMI
C15orf48
PPM1K
ETV7
PML
CD38
ADAR
TREX1
IGFBP2
G6PC
ZBP1
ALDH1A1
UBA7

SPSB1
GBP1
PARP10
GCA
IMPA2
PMAIP1
SNN
IFITM1
C4orf33
OAS2
IFI6
B4GALT5
CASP1
IFIT1
WARS
MAX
FFAR2
RNASE4
SERPING1
C1S
IL1RN
AMPH
IFIT2
APOL3
BUB1
HLA-E
KIAA0082
APOBEC3A
CEBPD
BLVRA
ANKRD22
TFEC
PRKD2
KIAA0082
COMMD3
TAP1
MSR1
TNFSF13B
MAFB
TXNIP
CCND3

Supplementary Table 2. List of anti-flu genes

BRD4
BST2
CCL5
CCL3
CCL4
MB21D1
CH25H
CRY1
CTR9
EIF2AK2
ELF1
HERC5
IFIT1
IFIT2
IFIT3
IFIT5
IFIT1B
IFITM2
IFITM1
IFITM3
IFITM10
IFITM1
IFITM2
IFITM3
IFITM5
IRF1
IRF7
ISG15
ISG20
LGALS3BP
MAFF
MAP3K14
MOV10
MX1
MX2
OAS1
OAS2
PAF1
PMM2
RNASEL
RSAD2
RTF1
SC02
SERPINE1
TBX3
TNFSF10
TRIM3
TRIM4
TRIM5

TRIM7
TRIM8
TRIM9
TRIM19
TRIM22
TRIM25
TRIM56
XAF1
ZAP70

Supplementary Table 3. Relative TLR expression in MoDC after different treatment conditions

symbol	log2FoldChange	baseMean	lfcSE	stat	pvalue	padj	condition
TLR3	3.04082054661727	315.755171415351	0.465778075708235	6.58663494911428	4.49906634905669e-11	4.68063581242934e-08	MOI0.02 vs Control
TLR7	1.38232995963052	233.760082485199	0.617738741428938	2.81917022910466	0.00481479762689811	0.0773181118365722	MOI0.02 vs Control
TLR8	0.443344464267065	1361.02443773807	0.422562166305436	1.05763421920085	0.290222235545246	0.677219850632489	MOI0.02 vs Control
TLR4	0.295636636250967	969.82227309137	0.330387096593614	0.904459309091697	0.365751901521992	0.736611787288139	MOI0.02 vs Control
TLR8-AS1	0.151270861246343	6.3427297313861	0.619756379935751	0.248829470982727	0.803492693389048	0.952304587778622	MOI0.02 vs Control
TLR1	0.131982205746097	680.487289018465	0.399886163809957	0.325189420250551	0.745037715348199	0.928974726597877	MOI0.02 vs Control
TLR9	0	0.186463402906586	0.213965018870988	0	1	NA	MOI0.02 vs Control
TLR2	-0.249893462442173	440.533911673885	0.360074646206924	-0.685624883586254	0.492949685870385	0.818391786554621	MOI0.02 vs Control
TLR10	-0.579484173884417	42.9027718601443	0.481721973178214	-1.22410557813551	0.220912402034178	0.612055487757933	MOI0.02 vs Control
TLR6	-0.685537766833045	333.114584360856	0.537553221051512	-1.24005010260494	0.214956863045778	0.604760809399604	MOI0.02 vs Control
TLR5	-0.828817162143339	9.86144185739866	0.623703304811676	-1.28271849991827	0.199590716136359	0.588101946985203	MOI0.02 vs Control
TLR3	3.13036923769003	315.755171415351	0.465796274756401	6.73330087046834	1.65856598726946e-11	8.33000469123438e-09	MOI0.2 vs Control
TLR7	2.3075662569975	233.760082485199	0.617491066181485	4.27852389401539	1.88136796902939e-05	0.000730850722653567	MOI0.2 vs Control
TLR8	0.881088946345194	1361.02443773807	0.422431948550553	2.09739822919584	0.0359583377203379	0.156806343981054	MOI0.2 vs Control
TLR9	0.129374855170082	0.186463402906586	0.213965018870988	0.434937169865404	0.663608033681306	NA	MOI0.2 vs Control
TLR1	0.105219401768066	680.487289018465	0.39996762459834	0.260094677796668	0.794790743192245	0.902170260014991	MOI0.2 vs Control
TLR10	0.0652508063285099	42.9027718601443	0.476216473867782	0.133398937974812	0.893877885275605	0.951765731824592	MOI0.2 vs Control
TLR4	-0.0658763132849698	969.82227309137	0.330669566857101	-0.191126316356708	0.848426630199961	0.929052993674343	MOI0.2 vs Control
TLR8-AS1	-0.145742469912415	6.3427297313861	0.623797979515845	-0.280622544813986	0.778999922329196	0.891991656346285	MOI0.2 vs Control
TLR2	-0.519289279272163	440.533911673885	0.360751854927352	-1.4367896156018	0.150777781944797	0.364673327616188	MOI0.2 vs Control
TLR6	-1.02118172558279	333.114584360856	0.537844253981147	-1.85747435827618	0.0632436959875521	0.21842856053375	MOI0.2 vs Control
TLR5	-1.62631480075674	9.86144185739866	0.62741776166654	-2.61531810525293	0.00891443884749135	0.0645322076608904	MOI0.2 vs Control
TLR7	2.41880902504492	233.760082485199	0.617568710993472	4.3119943613008	1.61788560959324e-05	0.00201406016271158	polyIC vs Control
TLR8	1.22721033049764	1361.02443773807	0.42244632408442	2.91351807397174	0.00357381148450342	0.0689437937374733	polyIC vs Control
TLR3	1.04138993323487	315.755171415351	0.468874461191208	2.34263770470104	0.0191479664867377	0.18594167843868	polyIC vs Control
TLR8-AS1	0.419593041088417	6.3427297313861	0.622084376401424	0.718890699048575	0.47220826763792	0.716503116798241	polyIC vs Control
TLR2	0.19339816684934	440.533911673885	0.360145693368514	0.544734322013271	0.585936255629876	0.790390416184367	polyIC vs Control
TLR9	0	0.186463402906586	0.213965018870988	0	1	NA	polyIC vs Control
TLR4	-0.0485596155968636	969.82227309137	0.33093292173148	-0.148006883511185	0.882337335820608	0.951902490949894	polyIC vs Control
TLR5	-0.120872220557354	9.86144185739866	0.622908468525795	-0.273274299075153	0.784642370056696	0.904886981928665	polyIC vs Control
TLR1	-0.360454630484001	680.487289018465	0.40060344103791	-0.894420213901513	0.371097101151805	0.643805508491756	polyIC vs Control
TLR10	-0.496145664947137	42.9027718601443	0.487696670703658	-1.0516656557051	0.292952973393357	0.580785034508786	polyIC vs Control
TLR6	-0.688683652848225	333.114584360856	0.537877786090874	-1.23720272677277	0.216011829468594	0.51249589447957	polyIC vs Control
TLR3	1.49369066183655	315.755171415351	0.467013750467002	3.2406630235112	0.00119252062364591	0.0808245078401066	ssON0.5 vs Control
TLR7	1.07447260431702	233.760082485199	0.617886460902203	2.46935365113971	0.0135357372769664	0.231268546383005	ssON0.5 vs Control
TLR8	0.987045375316752	1361.02443773807	0.422344758706928	2.35221436449241	0.0186620165856072	0.257327145944819	ssON0.5 vs Control
TLR10	0.301889414044293	42.9027718601443	0.473116702211204	0.637445919542108	0.523834425782516	0.786010364929511	ssON0.5 vs Control
TLR8-AS1	0.193571931545212	6.3427297313861	0.619068337839497	0.255928789396558	0.798005824023432	0.925085866210832	ssON0.5 vs Control
TLR9	0	0.186463402906586	0.213965018870988	0	1	NA	ssON0.5 vs Control

TLR2	-0.270341163575651	440.533911673885	0.360031422604979	-0.750411756563839	0.4530067524201	0.74766315347712	ssON0.5 vs Control
TLR5	-0.342265405533042	9.86144185739866	0.621816349844269	-0.420603781531951	0.674044431932546	0.866139821983724	ssON0.5 vs Control
TLR1	-0.362611973732186	680.487289018465	0.400163824463829	-0.908435107647667	0.363648384152119	0.691149320136746	ssON0.5 vs Control
TLR6	-0.397826546120566	333.114584360856	0.537402308834284	-0.72079684253075	0.471034518368805	0.757678891707252	ssON0.5 vs Control
TLR4	-0.562029006681708	969.82227309137	0.33090936076704	-1.6824389450047	0.0924837548410217	0.440848573332202	ssON0.5 vs Control
TLR7	3.220788880882	233.760082485199	0.617418676583411	5.61102040517175	2.01137043763929e-08	1.21807407129399e-06	MOI0.2_polyIC vs Control
TLR8-AS1	1.59596502980079	6.3427297313861	0.612913098297581	2.41210511038482	0.0158607082919204	0.0396469218431327	MOI0.2_polyIC vs Control
TLR8	1.48597979392089	1361.02443773807	0.422422346919665	3.5250558193106	0.000423393565406983	0.00212515499542676	MOI0.2_polyIC vs Control
TLR10	1.16720203948585	42.9027718601443	0.472100946812999	2.45019813120696	0.0142777625802486	0.0365201440092935	MOI0.2_polyIC vs Control
TLR3	1.14485119937844	315.755171415351	0.469192775132823	2.50251424691385	0.0123314662237962	0.0323998179880686	MOI0.2_polyIC vs Control
TLR2	0.513023879847566	440.533911673885	0.36000565500304	1.41525110355409	0.156994885811763	0.248460018163143	MOI0.2_polyIC vs Control
TLR9	0	0.186463402906586	0.213965018870988	0	1	NA	MOI0.2_polyIC vs Control
TLR4	-0.513106682520064	969.82227309137	0.331696308138343	-1.52838917582918	0.126415940679822	0.209705810299972	MOI0.2_polyIC vs Control
TLR5	-0.534014022620221	9.86144185739866	0.625361840750066	-0.732581265228048	0.463813861158773	0.572649911025248	MOI0.2_polyIC vs Control
TLR1	-1.14830379785956	680.487289018465	0.402001309006235	-2.86196142542504	0.00421028146184968	0.0135746712826553	MOI0.2_polyIC vs Control
TLR6	-2.4783659665817	333.114584360856	0.541038752187877	-4.65961654592351	3.16798985676827e-06	4.86442611527996e-05	MOI0.2_polyIC vs Control
TLR3	1.99355731092185	315.755171415351	0.466485282135152	4.34643325362123	1.38369131047571e-05	0.00280702350714343	MOI0.2_ssON0.5 vs Control
TLR7	1.06254735539798	233.760082485199	0.617888030616518	2.29647444271182	0.0216487688217244	0.354794014794461	MOI0.2_ssON0.5 vs Control
TLR10	0.915951023070411	42.9027718601443	0.469466053455209	1.93476653011168	0.0530189520352364	0.510532718379069	MOI0.2_ssON0.5 vs Control
TLR8	0.351212538802053	1361.02443773807	0.422604563522587	0.837577190255629	0.402268207882365	0.857438480219999	MOI0.2_ssON0.5 vs Control
TLR9	0.0416740551043146	0.186463402906586	0.213965018870988	0.201089078004096	0.840628922052501	NA	MOI0.2_ssON0.5 vs Control
TLR1	0.0304941466179177	680.487289018465	0.399935059675036	0.0753988523076199	0.939897372337967	0.993980400138984	MOI0.2_ssON0.5 vs Control
TLR4	-0.123810682459653	969.82227309137	0.330610899288326	-0.368310301800035	0.712641871883218	0.952389343296148	MOI0.2_ssON0.5 vs Control
TLR6	-0.286173451521566	333.114584360856	0.537363546820125	-0.512260130956934	0.608468964326536	0.926523425539839	MOI0.2_ssON0.5 vs Control
TLR8-AS1	-0.621911309717392	6.3427297313861	0.626701216013519	-1.19578374536005	0.231780960687907	0.781639953478903	MOI0.2_ssON0.5 vs Control
TLR5	-0.96868934362565	9.86144185739866	0.624194855172279	-1.44210169768646	0.149273686215509	0.70806336181378	MOI0.2_ssON0.5 vs Control
TLR2	-1.08786434745549	440.533911673885	0.361706595425651	-3.01429274811749	0.00257579129597003	0.109076447577664	MOI0.2_ssON0.5 vs Control
TLR7	1.69535349462855	233.760082485199	0.616527215942693	2.65373861492366	0.00796054648031297	0.0336301142252855	MOI0.02_polyIC vs MOI0.02
TLR10	1.14718260602619	42.9027718601443	0.48264044460366	2.38202280525918	0.0172178311347821	0.0587574632860534	MOI0.02_polyIC vs MOI0.02
TLR8-AS1	1.13113016770329	6.3427297313861	0.613739436972793	1.78597213885431	0.074103769360852	0.170081010280078	MOI0.02_polyIC vs MOI0.02
TLR2	1.06522108370289	440.533911673885	0.359877924985491	2.93018669792778	0.0033875840589263	0.0184522539523228	MOI0.02_polyIC vs MOI0.02
TLR8	1.02170296640472	1361.02443773807	0.42217023949553	2.42052405564105	0.0154981538354415	0.0544867178870368	MOI0.02_polyIC vs MOI0.02
TLR5	0.0133921775376044	9.86144185739866	0.628925984840877	0.0994907499463355	0.920748631935245	0.954643170288825	MOI0.02_polyIC vs MOI0.02
TLR9	0	0.186463402906586	0.213965018870988	0	1	NA	MOI0.02_polyIC vs MOI0.02
TLR4	-0.93102918495744	969.82227309137	0.33162296554425	-2.80614430615194	0.00501382329017328	0.0241396777524314	MOI0.02_polyIC vs MOI0.02
TLR1	-1.19589704050308	680.487289018465	0.401704799184545	-2.97612084348315	0.00291919807354219	0.0165245103619967	MOI0.02_polyIC vs MOI0.02
TLR3	-1.81278920739559	315.755171415351	0.463975861992986	-3.92038583095495	8.84073118425005e-05	0.00137709381463617	MOI0.02_polyIC vs MOI0.02
TLR6	-1.98828381688391	333.114584360856	0.541797388777619	-3.83481080921233	0.000125660980602512	0.00174992823822347	MOI0.02_polyIC vs MOI0.02
TLR6	0.747239563486973	333.114584360856	0.537559828950929	1.34908064547696	0.177311064187124	0.72832238758424	MOI0.02_ssON0.5 vs MOI0.02
TLR5	0.107720470716641	9.86144185739866	0.6263312201243	0.11699131496406	0.906866935873379	0.990318337155036	MOI0.02_ssON0.5 vs MOI0.02
TLR10	0.00142409989524935	42.9027718601443	0.488428121496925	0.00469592440270151	0.996253208190917	1	MOI0.02_ssON0.5 vs MOI0.02

TLR9	0	0.186463402906586	0.213965018870988	0	1	NA	MOI0.02_ssON0.5 vs MOI0.02
TLR1	-0.0957298490948378	680.487289018465	0.399909094876041	-0.23649471775337	0.813048805670914	0.986386924311597	MOI0.02_ssON0.5 vs MOI0.02
TLR4	-0.11135561554037	969.82227309137	0.330340813184197	-0.350473436300436	0.725983422294654	0.975460222328303	MOI0.02_ssON0.5 vs MOI0.02
TLR8-AS1	-0.299507494880069	6.3427297313861	0.621679959909944	-0.570275185334373	0.56849106865728	NA	MOI0.02_ssON0.5 vs MOI0.02
TLR2	-0.385865303774996	440.533911673885	0.361277314561381	-1.08204049419753	0.279234534934753	0.823452589852581	MOI0.02_ssON0.5 vs MOI0.02
TLR8	-0.543666011096079	1361.02443773807	0.42267910162896	-1.29982444155116	0.193661146446058	0.746878499288041	MOI0.02_ssON0.5 vs MOI0.02
TLR7	-1.21285103952885	233.760082485199	0.61761092423071	-2.41839769245227	0.0155890286120426	0.263366214312472	MOI0.02_ssON0.5 vs MOI0.02
TLR3	-2.17967429598386	315.755171415351	0.463525769473093	-4.70508168353187	2.5376433004915e-06	0.00202646677814875	MOI0.02_ssON0.5 vs MOI0.02
TLR8-AS1	1.74170749971321	6.3427297313861	0.615581408101237	2.61235172903898	0.00899216879869355	0.0472983795369186	MOI0.2_polyIC vs MOI0.2
TLR10	1.10195123315734	42.9027718601443	0.472596972188625	2.31147017510792	0.0208068987053017	0.0848914877501342	MOI0.2_polyIC vs MOI0.2
TLR5	1.09230077813652	9.86144185739866	0.631487906764294	1.9085959925684	0.0563142293010661	0.16812701250776	MOI0.2_polyIC vs MOI0.2
TLR2	1.03231315911973	440.533911673885	0.361012161548449	2.84590760919181	0.00442850416789298	0.0285132219622897	MOI0.2_polyIC vs MOI0.2
TLR7	0.913222623884502	233.760082485199	0.616249238469946	1.37181566827304	0.1701208283883	0.345811687844146	MOI0.2_polyIC vs MOI0.2
TLR8	0.604890847575701	1361.02443773807	0.422043796869701	1.42951588285702	0.152856013200472	0.323920645382604	MOI0.2_polyIC vs MOI0.2
TLR9	-0.109177845507946	0.186463402906586	0.213965018870988	-0.339270821065988	0.734405721887342	NA	MOI0.2_polyIC vs MOI0.2
TLR4	-0.447230369235094	969.82227309137	0.331813603952188	-1.33742500858409	0.181083945705752	0.359831083608892	MOI0.2_polyIC vs MOI0.2
TLR1	-1.25352319962763	680.487289018465	0.40200906958663	-3.12015173324073	0.00180757903468208	0.014975007661924	MOI0.2_polyIC vs MOI0.2
TLR6	-1.45718424099891	333.114584360856	0.541611608830973	-2.82980220304115	0.0046576789429841	0.0294611533468049	MOI0.2_polyIC vs MOI0.2
TLR3	-1.98551803831158	315.755171415351	0.464363795346851	-4.23758903329879	2.25932836478311e-05	0.00056062531607034	MOI0.2_polyIC vs MOI0.2
TLR10	0.850700216741901	42.9027718601443	0.469965833237243	1.79577910476573	0.0725296551842	0.799687922264296	MOI0.2_ssON0.5 vs MOI0.2
TLR6	0.735008274061229	333.114584360856	0.537943177128955	1.34571522501983	0.178394374372005	0.883302338020264	MOI0.2_ssON0.5 vs MOI0.2
TLR5	0.657625457131088	9.86144185739866	0.630413597574606	1.29708363401761	0.194602411990995	NA	MOI0.2_ssON0.5 vs MOI0.2
TLR4	-0.0579343691746829	969.82227309137	0.33072864370172	-0.177072765936727	0.859451233505107	0.989368063918425	MOI0.2_ssON0.5 vs MOI0.2
TLR1	-0.0747252551501484	680.487289018465	0.399942867078331	-0.184717357149326	0.85345073141661	0.989195753355623	MOI0.2_ssON0.5 vs MOI0.2
TLR9	-0.0877008000657677	0.186463402906586	0.213965018870988	-0.231449258177353	0.816965801320966	NA	MOI0.2_ssON0.5 vs MOI0.2
TLR8-AS1	-0.476168839804978	6.3427297313861	0.629054750039434	-0.925006693638161	0.354962425642296	NA	MOI0.2_ssON0.5 vs MOI0.2
TLR8	-0.529876407543141	1361.02443773807	0.422226214440362	-1.26027342574042	0.20757074299507	0.894420830350415	MOI0.2_ssON0.5 vs MOI0.2
TLR2	-0.568575068183325	440.533911673885	0.362707644906642	-1.57726860957682	0.114733732003107	0.849669934310594	MOI0.2_ssON0.5 vs MOI0.2
TLR3	-1.13681192676818	315.755171415351	0.461616956272598	-2.4214428923337	0.0154590298102579	0.572209730252958	MOI0.2_ssON0.5 vs MOI0.2
TLR7	-1.24501890159952	233.760082485199	0.616722677450186	-2.02405070959404	0.0429649344237274	0.72496263307506	MOI0.2_ssON0.5 vs MOI0.2
TLR8-AS1	1.74170749971321	6.3427297313861	0.615581408101237	2.61235172903898	0.00899216879869355	0.0472983795369186	MOI0.2_polyIC vs MOI0.2
TLR10	1.10195123315734	42.9027718601443	0.472596972188625	2.31147017510792	0.0208068987053017	0.0848914877501342	MOI0.2_polyIC vs MOI0.2
TLR5	1.09230077813652	9.86144185739866	0.631487906764294	1.9085959925684	0.0563142293010661	0.16812701250776	MOI0.2_polyIC vs MOI0.2
TLR2	1.03231315911973	440.533911673885	0.361012161548449	2.84590760919181	0.00442850416789298	0.0285132219622897	MOI0.2_polyIC vs MOI0.2
TLR7	0.913222623884502	233.760082485199	0.616249238469946	1.37181566827304	0.1701208283883	0.345811687844146	MOI0.2_polyIC vs MOI0.2
TLR8	0.604890847575701	1361.02443773807	0.422043796869701	1.42951588285702	0.152856013200472	0.323920645382604	MOI0.2_polyIC vs MOI0.2
TLR9	-0.109177845507946	0.186463402906586	0.213965018870988	-0.339270821065988	0.734405721887342	NA	MOI0.2_polyIC vs MOI0.2
TLR4	-0.447230369235094	969.82227309137	0.331813603952188	-1.33742500858409	0.181083945705752	0.359831083608892	MOI0.2_polyIC vs MOI0.2
TLR1	-1.25352319962763	680.487289018465	0.40200906958663	-3.12015173324073	0.00180757903468208	0.014975007661924	MOI0.2_polyIC vs MOI0.2
TLR6	-1.45718424099891	333.114584360856	0.541611608830973	-2.82980220304115	0.0046576789429841	0.0294611533468049	MOI0.2_polyIC vs MOI0.2
TLR3	-1.98551803831158	315.755171415351	0.464363795346851	-4.23758903329879	2.25932836478311e-05	0.00056062531607034	MOI0.2_polyIC vs MOI0.2

TLR10	0.850700216741901	42.9027718601443	0.469965833237243	1.79577910476573	0.0725296551842	0.799687922264296	MOI0.2_ssON0.5 vs MOI0.2
TLR6	0.735008274061229	333.114584360856	0.537943177128955	1.34571522501983	0.178394374372005	0.883302338020264	MOI0.2_ssON0.5 vs MOI0.2
TLR5	0.657625457131088	9.86144185739866	0.630413597574606	1.29708363401761	0.194602411990995	NA	MOI0.2_ssON0.5 vs MOI0.2
TLR4	-0.0579343691746829	969.82227309137	0.33072864370172	-0.177072765936727	0.859451233505107	0.989368063918425	MOI0.2_ssON0.5 vs MOI0.2
TLR1	-0.0747252551501484	680.487289018465	0.399942867078331	-0.184717357149326	0.85345073141661	0.989195753355623	MOI0.2_ssON0.5 vs MOI0.2
TLR9	-0.0877008000657677	0.186463402906586	0.213965018870988	-0.231449258177353	0.816965801320966	NA	MOI0.2_ssON0.5 vs MOI0.2
TLR8-AS1	-0.476168839804978	6.3427297313861	0.629054750039434	-0.925006693638161	0.354962425642296	NA	MOI0.2_ssON0.5 vs MOI0.2
TLR8	-0.529876407543141	1361.02443773807	0.422226214440362	-1.26027342574042	0.20757074299507	0.894420830350415	MOI0.2_ssON0.5 vs MOI0.2
TLR2	-0.568575068183325	440.533911673885	0.362707644906642	-1.57726860957682	0.114733732003107	0.849669934310594	MOI0.2_ssON0.5 vs MOI0.2
TLR3	-1.13681192676818	315.755171415351	0.461616956272598	-2.4214428923337	0.0154590298102579	0.572209730252958	MOI0.2_ssON0.5 vs MOI0.2
TLR7	-1.24501890159952	233.760082485199	0.616722677450186	-2.02405070959404	0.0429649344237274	0.72496263307506	MOI0.2_ssON0.5 vs MOI0.2

Supplementary Table 4. List of inflammasome genes

APAF1
BIRC2
BIRC3
CASP1
CASP2
CASP4
CASP5
CASP9
CRADD
RIPK2
BCL10
NOL3
DLG5
NOD1
NLRP1
CARD8
PYCARD
CARD10
QRICH1
NLRC4
CARD18
NOD2
CARD9
CARD14
CARD11
CARD6
CARD16
CARD17
CASP12
BGN
C21orf2
CD14
CHAD
CPN2
DCN
EPYC
ECM2
FLII
FMOD
LRRC32
GP1BA
GP5
IGFALS
ISLR
LRCH4
LUM
CD180
CIITA
NTRK3

OMD
OGN
OMG
PKD1
PPP1R7
PRELP
RABGGTA
RSU1
SLIT1
SLIT3
SNRPA1
TLR1
TLR2
TLR3
TLR4
TLR5
TPBG
PXDN
SHOC2
ANP32A
LGR5
MFHAS1
SLIT2
LRIG2
TRIL
LRRC37A
LRRC23
LRRC17
TLR6
LRRN2
NXF1
ANP32B
CNTRL
KERA
NISCH
PHLPP2
LRCH1
PHLPP1
LRRC8B
SCRIB
ANP32D
ANP32C
LRRC6
FLRT3
FLRT1
GPR124
TSKU
LRIG1
LRRTM2
SLITRK5

LRIT1
OPTC
TLR7
TLR8
TLR9
LRRN3
ASPN
LRRC49
LRRC8D
LRRC1
LRRC36
LGR4
PIDD
LRRC59
LRRC40
CEP72
ERBB2IP
NXF5
NXF2
LRRC8A
LRTM1
AMIGO1
LRRC47
CNOT6
LRFN2
LRRC7
ISLR2
LRFN1
LRCH2
LRRN1
LRRC4C
RXFP1
LGR6
LRRC4
RTN4R
LRRC61
LRRC2
CEP97
ZYG11B
LRRK1
PODNL1
LRRTM4
LRRC8E
LRRC27
LRRC3
ANP32E
TLR10
LRRC48
DNAL1
C10orf11

LRRIQ1
NLRC5
SLITRK6
LRRC8C
SLITRK2
LINGO1
LRRCC1
LRRC46
LRSAM1
NLRP12
LRRC4B
LRRC37B
STK11IP
ELFN2
SLITRK1
VASN
LRRC56
LRRC58
LRRC3B
LRG1
LRRK2
LRIG3
RXFP2
LRR1
LRRC28
DNAAF1
LRRC38
LRRIQ3
PODN
LRRC39
LRRC15
LRGUK
PXDNL
SLITRK4
XRRA1
LRFN5
RTN4RL1
CHADL
LINGO2
FBXO39
LGI4
GPR125
LGI3
LRRC55
LRTOMT
LRRC63
LRWD1
CNOT6L
LRRC43
LRRC57

LRRC68
LRRC67
LRRC30
LINGO4
LRRC66
LRIT2
LRRC9
LRRIQ4
LRIT3
LRRTM1
LRRTM3
AMIGO2
LRRC37A3
LRRC33
LRRC10
AMIGO3
LRRC10B
ELFN1
LRRD1
LRRC52
LRRC24
LRRC37A2
LRRC18
LINGO3
LOC647020
PRAMEL
LRTM2
ANP32AP1
NXF2B
LRRC70
LRRC69
LRRC53
LRRC3C
LOC100506049
LOC100507326
NAIP
TEP1
NLRP2
NLRX1
NLRP3
NLRP13
NLRP8
NLRP5
NLRP4
NLRP6
NLRC3
NLRP7
NLRP11
NLRP9
NLRP10

NLRP14
LOC100509323
IFI16
MEFV
MNDA
AIM2
PYHIN1
PYDC2
PYDC1
LOC646377
XIAP
BIRC5
BIRC6
BIRC7
BIRC8
CASP3
CASP6
CASP7
CASP8
CASP10
CASP14
CASP14L
CFLAR
MALT1
LOC643733
